# PARP1-targeted fluorescence molecular endoscopy as novel tool for early detection of esophageal dysplasia and adenocarcinoma

**DOI:** 10.1186/s13046-024-02963-7

**Published:** 2024-02-21

**Authors:** Sabrina Marcazzan, Marcos J. Braz Carvalho, Nghia T. Nguyen, Julia Strangmann, Julia Slotta-Huspenina, Anna Tenditnaya, Markus Tschurtschenthaler, Jonas Rieder, Andrea Proaño-Vasco, Vasilis Ntziachristos, Katja Steiger, Dimitris Gorpas, Michael Quante, Susanne Kossatz

**Affiliations:** 1 II. Medizinische Klinik, TUM School of Medicine and Health, Klinikum Rechts der Isar at Technical University of Munich, Munich, 81675 Germany; 2 Institute of Biological and Medical Imaging, Helmholtz Zentrum München, 85764 Neuherberg, Germany and Chair of Biological Imaging at the Central Institute for Translational Cancer Research (TranslaTUM), TUM School of Medicine and Health, Technical University of Munich, Munich, 81675 Germany; 3 Department of Nuclear Medicine, TUM School of Medicine and Health, Klinikum Rechts der Isar at Technical University of Munich, Munich, 81675 Germany; 4 Central Institute for Translational Cancer Research (TranslaTUM), TUM School of Medicine and Health, Technical University of Munich, Munich, 81675 Germany; 5 Department of Medicine II (Gastroenterology, Hepatology, Endocrinology, and Infectious Diseases), Freiburg University Medical Center, Faculty of Medicine, University of Freiburg, Freiburg, 79106 Germany; 6 Institute of Pathology, TUM School of Medicine and Health, Technical University of Munich, Munich, 81675 Germany; 7 Department of Chemistry, TUM School of Natural Sciences, Technical University of Munich, Munich, 85748 Germany; 8 Comparative Experimental Pathology (CEP) and IBioTUM tissue biobank, TUM School of Medicine and Health, Technical University of Munich, München, 81675 Germany; 9 Division of Translational Cancer Research, German Cancer Research Center (DKFZ) and German Cancer Consortium (DKTK), Heidelberg, 69120 Germany; 10 Chair of Translational Cancer Research and Institute of Experimental Cancer Therapy, TUM School of Medicine and Health, Klinikum rechts der Isar at Technical University of Munich, Munich, 81675 Germany; 11 Present Address: Clinical Radiology, Medical School OWL, Bielefeld University, Bielefeld, 33615 Germany

**Keywords:** Dysplasia, Esophageal Adenocarcinoma, PARP1, Fluorescence Molecular Endoscopy, Fluorescence Imaging, Animal Models

## Abstract

**Background:**

Esophageal cancer is one of the 10 most common cancers worldwide and its incidence is dramatically increasing. Despite some improvements, the current surveillance protocol with white light endoscopy and random untargeted biopsies collection (Seattle protocol) fails to diagnose dysplastic and cancerous lesions in up to 50% of patients. Therefore, new endoscopic imaging technologies in combination with tumor-specific molecular probes are needed to improve early detection. Herein, we investigated the use of the fluorescent Poly (ADP-ribose) Polymerase 1 (PARP1)-inhibitor PARPi-FL for early detection of dysplastic lesions in patient-derived organoids and transgenic mouse models, which closely mimic the transformation from non-malignant Barrett’s Esophagus (BE) to invasive esophageal adenocarcinoma (EAC).

**Methods:**

We determined PARP1 expression via immunohistochemistry (IHC) in human biospecimens and mouse tissues. We also assessed PARPi-FL uptake in patient- and mouse-derived organoids. Following intravenous injection of 75 nmol PARPi-FL/mouse in L2-*IL1B* (*n* = 4) and L2-*IL1B*/*IL8*Tg mice (*n* = 12), we conducted fluorescence molecular endoscopy (FME) and/or imaged whole excised stomachs to assess PARPi-FL accumulation in dysplastic lesions. L2-*IL1B*/*IL8*Tg mice (*n* = 3) and wild-type (WT) mice (*n* = 2) without PARPi-FL injection served as controls. The imaging results were validated by confocal microscopy and IHC of excised tissues.

**Results:**

IHC on patient and murine tissue revealed similar patterns of increasing PARP1 expression in presence of dysplasia and cancer. In human and murine organoids, PARPi-FL localized to PARP1-expressing epithelial cell nuclei after 10 min of incubation. Injection of PARPi-FL in transgenic mouse models of BE resulted in the successful detection of lesions via FME, with a mean target-to-background ratio > 2 independently from the disease stage. The localization of PARPi-FL in the lesions was confirmed by imaging of the excised stomachs and confocal microscopy. Without PARPi-FL injection, identification of lesions via FME in transgenic mice was not possible.

**Conclusion:**

PARPi-FL imaging is a promising approach for clinically needed improved detection of dysplastic and malignant EAC lesions in patients with BE. Since PARPi-FL is currently evaluated in a phase 2 clinical trial for oral cancer detection after topical application, clinical translation for early detection of dysplasia and EAC in BE patients via FME screening appears feasible.

**Supplementary Information:**

The online version contains supplementary material available at 10.1186/s13046-024-02963-7.

## Introduction

In this study, we investigated an approach for improved early detection of esophageal dysplasia and cancer using the DNA repair enzyme Poly (ADP-ribose) Polymerase 1 (PARP1) as imaging biomarker. Esophageal cancer is the eighth most common cancer globally with more than 450,000 newly diagnosed cases and more than 400,000 deaths annually. Of the two histological subtypes, esophageal squamous cell carcinoma (ESCC) remains overall more common, but the incidence of esophageal adenocarcinoma (EAC) has increased at a rate of 4 to 10% annually in regions of the Western World. This is an increase greater than that of any other cancer [1], along with a very poor prognosis and median survival of less than one year. EAC development is characterized by a classic inflammation-induced metaplasia-dysplasia-carcinoma sequence and is strongly linked to a predisposing condition called Barrett’s esophagus (BE). The incidence of BE is continuously rising, e.g., as a result of increasing obesity rates and long-term acid reflux disease [2, 3]. Since BE patients are at an increased risk for EAC, white light endoscopy (WLE) and biopsy surveillance are recommended to monitor the development of dysplasia and progression to EAC [4]. Current practice recommends surveillance every 2–5 years and consists of the collection of 4-quadrant biopsies every 1–2 cm across the entire length of the BE lesion, which is labor-intensive, costly, time-consuming, prone to sampling error, and highly invasive [5–9]. Furthermore, EAC does not present strong WLE optical contrast and WLE is limited to the mucosal surface [10]. As a result, even in combination with random biopsy collections, the detection miss-rate of dysplasia reaches 57% [11]. Recently, large population-based studies reported rates of progression from non-dysplastic BE to cancer of 0.10—0.13% per year [12, 13]. Hence, the current biopsy surveillance practice puts an enormous burden on BE patients and health care systems and still fails to reliably diagnose early EAC cases [8, 14]. Hence, a non-invasive, sensitive and specific method to identify in particular early malignant lesions (i.e. dysplasia) in BE patients is urgently needed.

Two commonly applied non-invasive imaging methods of the esophagus are WLE (wide-field scale) and cross-sectional imaging (microscopic scale). Nevertheless, although these approaches are reliant on detecting structural and morphological changes in the tissue that are typical for the development of EAC, they cannot assess the underlying molecular changes which initiate and drive disease progression. The success of morphological imaging is highly dependent on the experience of the performing physician and shows high inter- and intra-observer variability [15]. The addition of contrast agents to wide-field endoscopy to create detectable color differences between malignant and non-malignant tissue has been explored with non-specific agents, such as acetic acid, Lugol dye [16, 17], methylene blue [18, 19] and indocyanine green (ICG) [20, 21]. However, their low sensitivity and specificity limit their ability to provide additional diagnostic information [15], mainly attributed to the lack of a molecular target.

Optical molecular imaging (OI) using targeted fluorescent probes has the potential to improve early detection of epithelial tumors based on the presence of biomarkers overexpressed in tumor cells. During endoscopy in EAC surveillance, OI could significantly improve differentiation of malignant from non-malignant lesions. This type of optical contrast could be further used for surgical navigation, for post-surgery follow-up and ex vivo margin detection.

We have previously developed an OI approach for early detection of oral cancer that targets the DNA repair enzyme PARP1. PARP1 plays a central role in initiating the DNA damage repair response (DDR), especially for single-strand breaks [22, 23]. PARP1 expression is strongly increased in many different tumor types compared with healthy adult tissue for a number of reasons, including the high mutational burden and genomic instability of tumor cells and their increased proliferative activity and DNA content [24–32]. We recently showed that an upregulation of PARP1 also occurred in EAC compared with the normal tissue [33]. A potential role of PARP1 in BE pathogenesis has also been suggested previously [34]. Regarding the role of PARP1 in the other gastrointestinal tumors, we refer the readers to the review authored by Martin-Guerrero et al. [35].

Certain tumor types are vulnerable to inhibition of PARP1-mediated DNA repair as monotherapy or in combination with other treatments. This has led to the development of small molecule PARP inhibitors (PARPis): 4 of whom have been FDA and/or EMA approved with treatment indications in breast, ovarian, pancreatic and prostate cancer [36–39]. The olaparib-based fluorescent derivate PARPi-FL represents the only available clinically validated PARPi that has been conjugated to a fluorophore. PARPi-FL was recently clinically translated for oral cancer detection after topical application, following extensive preclinical characterization [33, 40–42].

In this study, we aim to investigate the potential of PARPi-FL for early detection of EAC and to differentiate early malignant from non-malignant disease stages, as this poses the current clinical challenge. We determined PARP1 expression in BE/EAC patient samples and in our L2-*IL1B* and L2-*IL1B/IL8* transgenic (Tg) mouse models of BE used for preclinical imaging [43, 44]. These models mimic the histologic progression from dysplasia to EAC observed in patients, based on chronic inflammation in the esophagus [45, 46]. Due to the age-dependent progression from BE (by 6 months) to EAC (older age) [46, 47], these mouse models have proved to be useful to evaluate strategies for early detection and diagnosis. Indeed, we showed a higher PARP1 expression in patient samples and mice with dysplastic tissues. Both patient-derived and murine organoids showed nuclear PARPi-FL accumulation and PARP1 expression. We were able to detect PARPi-FL lesion-specific uptake in dysplastic lesions of L2-*IL1B* mice by imaging of excised specimens. Finally, we also employed a custom-made endoscope for fluorescence molecular endoscopy (FME) [43, 44] of PARPi-FL in L2-*IL1B*/*IL8Tg* mice [46] to mimic the EAC detection by endoscopic screening in patients and we were able to detect PARPi-FL lesion-specific accumulation in dysplastic lesions, suggesting that PARPi-FL-targeted endoscopy may have a high translational potential for investigation in the clinical setting.

## Methods

### Animals

Mice overexpressing human *IL1B* (L2-*IL1B* mice) under the control of EBV-L2 were generated as described previously [45]. L2-*IL1B* mice were then backcrossed with C57BL/6 J mice. L2-*IL1B*/*IL8*Tg mice were obtained by breeding *IL8* mice with L2-*IL1B* mice as reported previously [46]. Due to their accelerated inflammatory and dysplastic phenotype [46], L2-*IL1B/IL8*Tg mice were used for endoscopic imaging while L2-*IL1B* mice were used for preliminary imaging experiments only (“Ex vivo wide-field imaging of mouse stomach”). After weaning at 21 days, mice were genotyped and fed with water and standard food or high-fat content diet (Ssniff, Germany) *ad libitum.* Wild-type (WT) mice were fed with water and standard food.

All animal experiments were performed following protocols approved by the Regierung von Oberbayern (ROB 55.2–2532.Vet_02-15–29, Vet_02-20–69, and Vet_02-16–24) according to the German Animal Welfare Act and Ethical Guidelines of the Klinikum rechts der Isar, Technical University of Munich (TUM).

### Patient tissues

Formalin-fixed and paraffin-embedded (FFPE) esophageal tissues from 14 patients who underwent endoscopic submucosal resection were used for this study. FFPE blocks were provided from the BarrettNET registry (ethical approval n°5291/12). Human specimens were fixed in a 10% neutral-buffered formalin solution for at least 48 h and routinely processed for histology. Samples were dehydrated overnight and embedded in paraffin. Subsequently, 2-µm-thick sections were prepared using a rotary microtome (RM2245, Leica Biosystems, Germany). Hematoxylin and eosin (HE) staining were performed according to a standard protocol. A HE-stained section per each patient sample was used for histopathological annotation by an experienced pathologist (JSH) using the following categories: normal squamous epithelium, BE, BE without intraepithelial neoplasia (IEN), BE with low-grade IEN, BE with high-grade IEN, EAC.

### Immunohistochemistry (IHC) for PARP1 and quantification

We analyzed PARP1 expression in patient samples (*n* = 14) and in stomach and esophagus from L2-*IL1B* mice of different age (6, 9 and 12 months; *n* = 2–3 per group) and wild-type (WT) mice (*n* = 3). Mouse tissues were formalin-fixed overnight, dehydrated, paraffin-embedded and 3 μm tissue sections were obtained for IHC. The staining for PARP1 was performed on human tissues using a Bond RXm system (Leica Biosystems, Germany, all reagents from Leica) with a primary mouse monoclonal antibody against PARP1 (clone 142, #66,520–1-Ig, Proteintech, US dilution 1:200). Briefly, slides were deparaffinized using deparaffinization solution, pretreated with Bond™ Epitope retrieval solution 1 (AR9961, Leica Biosystems) (corresponding to citrate buffer, pH6) for 30 min. Antibody binding was detected with a Bond™ polymer refine detection kit (DS9800, Leica Biosystems) and visualized with diaminobenzidine (DAB) as a dark brown precipitate. Counterstaining was done with hematoxylin. All slides were digitized (40 × magnification) using a brightfield slide scanner (Aperio AT2, Leica Microsystems, Germany). The PARP1 IHC on mouse tissues was performed by following the same procedure using an anti-PARP1 rabbit antibody (13,371–1-AP Proteintech, US; dilution 1:200).

For quantification of PARP1 expression, we followed our previously reported method using a color deconvolution and thresholding macro in ImageJ/Fiji software to determine the relative PARP1 positive tissue area (% PARP1 pta) [33].

In patient tissues, PARP1 expression was analyzed within each category listed above, while areas containing foveolar mucosa and salivary glands were not included in the analysis. An average of 5–7 20 × fields of view per patient per category was analyzed if the category was present in each sample.

The slides from mouse stomachs were also scanned (40 × magnification) and images were taken using an Aperio T2 Slide Scanner (Leica Microsystems, Germany) from the whole squamocolumnar junction (SCJ) of WT mice (3 to 5 fields of at 20X) and L2-*IL1B* mice (4 to 10 fields at 20x, depending on the SCJ extension). Data were plotted as means ± Standard Error of the Mean (SEM) and single values per each field.

### Organoid culture

Organoids were obtained from esophageal biopsies of two patients (after ethical approval within the BarrettNET clinical trial n° 5291/12) who presented BE/dysplasia and the cardia of L2-*IL1B* mice of different age groups (6 to 9 months old; tot *n* = 4 mice) according to a modified protocol reported by Pastula et al. [48]. Briefly, 1–2 mm tissue pieces were incubated with Accutase (Thermo Fisher Scientific Inc., US) for 15–20 min at room temperature (RT) with gentle shaking. EDTA-PBS without calcium and magnesium (DBPS, 2 mM) was then added and the sample was put on ice for 15 to 45 min. After that, PBS containing 10% FBS and 1% penicillin streptomycin was added and the sample was centrifuged at 300–500 Relative Centrifugal Force (RCF) for 7–10 min at 4 °C. After centrifugation, the pellet was resuspended in Matrigel (n. 356,231, Corning, US) in a 24-well plate (50 μl/well) and L-WRN cell conditioned medium plus growth factors specific for murine or human BE organoids was then added as reported previously [48]. Organoids were maintained at 37 °C in a humidified atmosphere of 5% CO_2_ and 95% air. Media was changed every 2–3 days and organoids were passaged after 7–10 days: the passage of human organoids was performed with additional digestion steps by using cell recovery solution (Corning, US) and TrypLE (Thermo Fisher Scientific Inc., US). All experiments were performed within passages 3 to 7.

### Synthesis of PARPi-FL

The fluorescent imaging agent PARPi-FL used for in vitro and in vivo experiments was synthesized as described previously [49]. For in vivo experiments, PARPi-FL was formulated as single animal dose of 75 nmol PARPi-FL in 30%PEG300/PBS congruent to the dose used in previous studies in mice [33, 50].

### PARPi-FL staining and confocal imaging of living organoids

After dissolving Matrigel using Cell Recovery Solution (Corning, US) and washing steps as in our previous publication [44], murine and human organoids (passage 4, 6 and 7) were incubated with 0.5 μM PARPi-FL for 10 min (in the dark and on ice). Afterwards, cold PBS (4 °C) was added before gentle centrifugation at 4 °C at 200 RCF to carefully remove excess of PARPi-FL. Matrigel (no. 356231, Corning, US) was then added and kept on ice until cells were pelleted on 8-well chamber slides (Ibidi, Cat. No.: 80827) before imaging using the Leica SP8 confocal microscope (Leica Microsystems, Germany). For the imaging, a total number of 10 human organoids (2 patients) and 17 murine organoids (4 mice; 3–5 organoids per mouse) were used.

### Organoid histology and IHC

For fixation of organoids, we adapted a previously published protocol [51]. Briefly, organoids were plated on glass cover slips, fixed in 4% paraformaldehyde (PFA) for 30 min, washed with PBS and processed for paraffin-embedding. For HE and PARP1 staining, 2.5 µm sections were cut and stained for PARP1 as described in “IHC for PARP1”.

### Ex vivo wide-field imaging of mouse stomach

Fifteen-to-eighteen-month-old L2-*IL1B* mice (*n* = 4) were intravenously (i.v.) injected with 75 nmol of PARPi-FL and sacrificed 1 h post-injection (p.i.). Two PBS-injected 19-month-old WT mice were used as negative controls. The stomach with esophagus was collected and ex vivo wide-field imaging was performed using a Leica M205 FCA fluorescence stereo microscope using an exposure time of 7 ms. PARPi-FL was detected in the FITC channel (Exc.: 470 nm/Em.: 525 nm), while tissue autofluorescence was detected in the tdTomato channel (Exc.: 545 nm/Em.: 620 nm). After imaging, all organs were fixed overnight in 4% PFA at 4° C.

Quantification of the PARPi-FL signal was performed on the 16-bit FITC channel images using ImageJ/Fiji software [52] First, regions of interest (ROIs) were manually drawn to outline macroscopically visible lesions localized at the SCJ in the brightfield (BF) images of the stomach of L2-*IL1B* mice. The mean fluorescence intensity (MFI) was then determined from 18 to 27 lesion ROIs per mouse. We calculated tumor-to background ratios (TBR) as in our previous publications [43, 44, 53]. Briefly, three ROIs per mouse manually drawn in the area of adjacent normal stomach were considered as background ROIs and the MFI from the lesion ROIs was divided by the MFI from background ROIs. Results are shown as TBR of single lesion ROI from each mouse and mean ± SEM.

We generated overlay images of the BF with the 16-bit FITC channel after autofluorescence subtraction (tdTomato channel) using the Image Calculator subtraction command of ImageJ/Fiji.

### Fluorescence Molecular Endoscopy (FME)

Male and female L2-*IL1B*/*IL8*Tg mice of different age groups (6–9 and 14–15 months old; *n* = 4–5 per group; *n* = 12 total) fed with standard diet (6–9 and 14–15 months old; *n* = 9) and L2-*IL1B*/*IL8*Tg mice fed with high-fat diet/HFD (7–9 months old; *n* = 3) were i.v. injected with 75 nmol PARPi-FL. Fifteen-month-old L2-*IL1B*/*IL8*Tg mice (*n* = 3) were used as PBS-injected controls to evaluate the specificity of the PARPi-FL signal detected by our system. Mice were sacrificed 1 h p.i. and FME was performed using a custom-made endoscopic system optimized for small animal imaging [43, 44]. Briefly, the system consists of a flexible fiberscope with 0.8 mm outer diameter (Schölly Fiber Optics GmbH, Germany) coupled into a highly sensitive electron-multiplying charge-coupled device (EMCCD; DV897DCS-BV, Andor Technology, Northern Ireland) with a long-pass (ET520LP, Chroma Technology, US) and a band-pass (D535/40, Chroma Technology, US) filter for fluorescence detection. A laser diode at 462 nm (1400 mW, Thorlabs, US) was used for excitation. The system software developed by our group allowed hardware control of the system, acquisition of the fluorescence channel and generation and storage of videos in AVI file format.

For the quantification of PARPi-FL, fluorescence signals of each lesion (esophageal regions with high fluorescence signal: lesion ROIs) and background ROIs (surrounding esophageal tissue with low fluorescence) were manually segmented using ImageJ/Fiji and the MFIs from at least 2–3 consecutive endoscopy frames were averaged. One to fourteen lesion ROIs per mouse were used to quantify fluorescence signal in PARPi-FL-injected L2-*IL1B*/*IL8*Tg mice. The fluorescence signal in PBS-injected *IL1B*/*IL8*Tg mice was quantified as well based on 2 to 4 lesion ROIs selected in each mouse. TBR of PARPi-FL-injected mice was calculated as in our previous publications [43, 44] by dividing the MFI from lesion ROI with averaged MFI from background ROIs.

After FME, the stomach plus esophagus and organs were collected and quantification of ex vivo wide-field imaging was performed as described in “Ex vivo wide-field imaging of mouse stomach”. To calculate TBRs, 10 to 23 lesion ROIs and 3 background ROIs were considered per mouse (n = 12 PARPi-FL-injected and *n* = 3 PBS-injected mice). Quantification of FME and wide-field imaging was performed blinded to PARPi-FL injected/non-injected. Data are plotted as single values per lesion ROI or as mean ± SEM.

### Macroscopic and histopathologic scoring of disease stage

Macroscopic scoring of disease stage for each mouse was carried out by evaluating SCJ coverage by lesions (score 0–4) and size of lesions (0–4) on excised tissue according to a method we previously reported [46]. The microscopic scoring was carried out on HE-stained FFPE sections of the SCJ and performed by an expert gastroenterologist (MQ), who evaluated the grade of inflammation, metaplasia, and dysplasia (0–4) as previously reported [46]. For the fluorescence quantification by endoscopy and ex vivo wide-field imaging, the macroscopic score and dysplasia grade of each mouse were averaged as in our previous work [46] and the following final combined score (score_comb_) was obtained: score_comb_ 1 (low score): mean less or equal to 2; score_comb_ 2 (middle score): mean between 2 and 2.5; score_comb_ 3 (high score): mean > 2.5.

### Confocal microscopy of PARPi-FL

After fixation in 4% PFA, half of the stomach and esophagus were placed in 15% sucrose solution in PBS for 3–4 h at 4° C, followed by 30% sucrose solution in PBS overnight. Tissues were then embedded in Optimal Cutting Temperature (OCT) compound and blocks were placed at -80 °C. For cutting, blocks were transferred to -20 °C the day before and 7 µm sections were cut with a cryotome (Thermo Fisher Scientific Inc., US). Slides were allowed to dry overnight or at 37 °C for 1 h before storage at -20 °C. For imaging, slides were thawed, washed with PBST (PBS 0.03% Triton X-100) and mounted with mounting medium containing DAPI (Vectashield, Vector Laboratories, US). Slides were stored at 4 °C protected from the light before image acquisition with a Leica SP8 confocal microscope (Leica Microsystems, Germany). For the detection of the PARPi-FL signal, the AlexaFluor 488 (AF488) channel was used.

Quantification of PARPi-FL signal in the stomach was performed with ImageJ/Fiji software with a threshold manually set from 25 to 255 on three fields of the SCJ at 40 × magnification, measuring the percentage of the fluorescent area of each field. Results are represented as single plotted values per each field and mean ± SEM.

### Statistical analysis

Data from fluorescence microscopy, ex vivo wide-field imaging, endoscopy, and PARP1 IHC on mice were analyzed with unpaired two-tailed t-test or one-way analysis of variance (ANOVA) followed by *post-hoc* Tukey test or Dunnett’s multiple comparisons test. Kruskal–Wallis test with Dunn’s correction for multiple comparison was used to analyze the PARP1 expression within different regions of patient samples. All statistical analyses were performed with GraphPad Prism (GraphPad software, US). Results with *p*-values *p* < 0.05 were considered statistically significant.

## Results

### PARP1 expression increased with occurrence of IEN in the BE-EAC transition

**Fig. 1:**
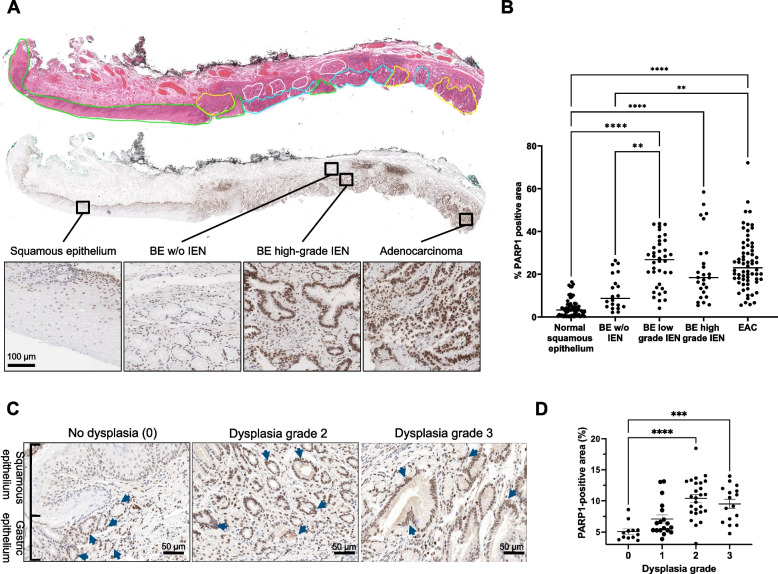
PARP1 expression along the BE to EAC transition in patient samples and in the L2-*IL1B* mouse model. **A** Human endoscopic submucosal resection samples (*n* = 14) were annotated according to their histopathology stage on HE-stained sections and PARP1 expression was analyzed within each category. In the displayed example, normal squamous epithelium (green), BE without high-grade Intraepithelial Neoplasia/IEN (white), BE with IEN (blue) and EAC (yellow) were present. **B** Quantification of the % PARP1 positive tissue area (pta) of all recorded 20 × fields-of-view and the mean value per category. * *p* < 0.05, ** *p* < 0.01, **** *p* < 0.0001 (Kruskal–Wallis-Test with Dunn’s correction for multiple comparisons). **C** Representative IHC images from the squamocolumnar junction (SCJ) of WT and L2-*IL1B* mice. In L2-*IL1B* mice, epithelial dysplastic cells (blue arrows) and inflammatory lymphocytes (orange arrows) were positive for PARP1 compared with WT mice, which did not present dysplastic epithelium and/or inflammation.** D** Quantification of PARP1 expression in murine SCJ tissues as % pta. PARP1 was significantly more expressed in dysplasia lesions graded 2 (*p* < 0.0001) and 3 (*p* = 0.0006) than normal areas without dysplasia (grade 0). Data are represented as single individually plotted values per field (3 WT mice and 2 to 4 L2-*IL1B* mice per group) and mean ± SEM. *** p < 0.001 and **** *p* < 0.0001 by one-way ANOVA followed by *posthoc* Tukey’s test

We analyzed PARP1 expression in 14 endoscopic submucosal resection samples, based on a classification into the following categories: normal squamous epithelium (NSE), BE without IEN, BE with low-grade IEN, BE with high-grade IEN, and EAC. Each sample displayed a mix of different histopathological subtypes, which showed distinct PARP1 expression patterns (Fig. 1A, Fig. S1). Quantification of PARP1 within the distinct subtypes revealed low PARP1 expression in areas of NSE (4.3 ± 4.3% PARP1 pta) and a non-significant (*p* = 0.0504) increase to 11.5 ± 8.1% PARP1 pta in areas of BE without IEN. In contrast, PARP1 expression in BE with low-grade IEN was significantly increased to 25.6 ± 11.3% pta compared with NSE (*p* < 0.0001) and BE without IEN (*p* = 0.002). BE with high-grade IEN and EAC showed comparable values of 22.7 ± 15.5% pta and 25.3 ± 12.8% pta, respectively (Fig. 1B). Hence, dysplastic and EAC lesions showed a distinctly higher PARP1 expression than NSE and BE without dysplasia, qualifying PARP1 as an early detection biomarker.

### PARP1 expression correlated with grade of dysplasia in L2-*IL1B* mice

We have previously shown that the L2-*IL1B* mouse model closely reflects the BE to EAC transition seen in humans [45]. Here, we characterized PARP1 expression in the stages of disease development to investigate if PARP1 can be used as early imaging biomarker and if the PARP1 expression pattern is similar to the human samples. We performed PARP1 IHC in L2-*IL1B* mice with different degrees of dysplasia and mice without dysplasia (WT mice). PARP1 was expressed only by cells of the normal gastric glandular epithelium (blue arrows) and the basal layer of esophageal squamous epithelium in mice without dysplasia (Fig. 1C). In L2-*IL1B* mice, PARP1 was also expressed by dysplastic BE epithelium proliferating at the SCJ and inflammatory lymphocytes recruited into the area. The percentage of PARP1% pta of SCJ tissue increased with the degree of dysplasia of L2-*IL1B* mice, with significantly higher PARP1 expression in dysplasia grades 2–3 than in grade 0 (no dysplasia), closely reflecting the expression pattern observed in human samples (Fig. 1D).

### PARPi-FL bound to human and murine BE organoids

**Fig. 2:**
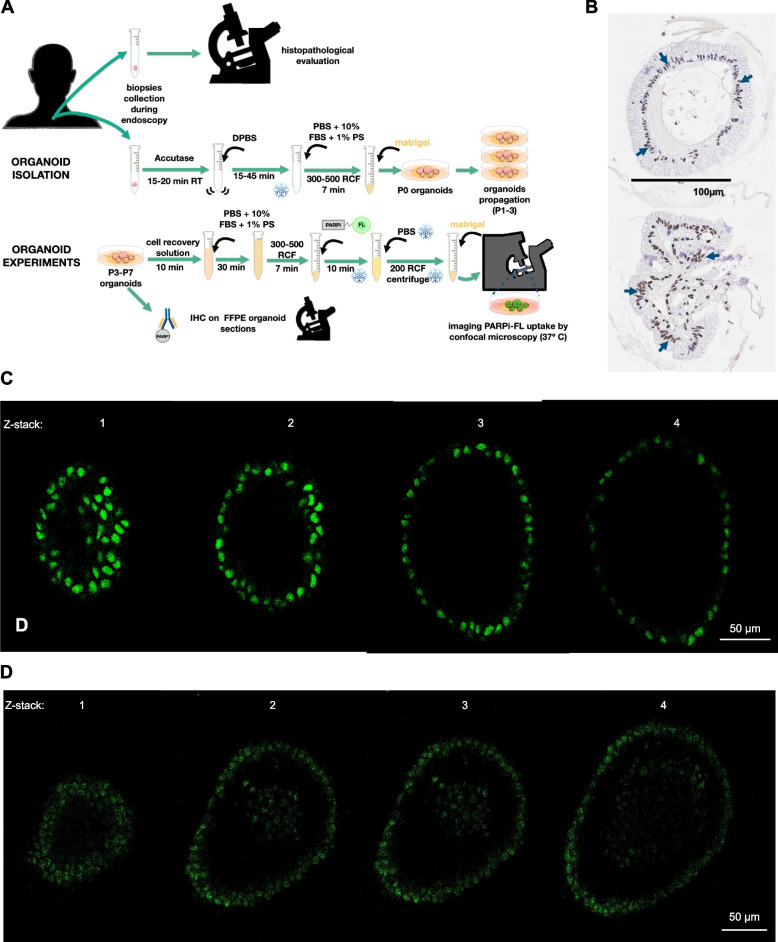
PARP1 expression and PARPi-FL binding in human and murine organoids. **A** Schematic of procedure of the isolation of organoids from patient biopsies and staining with PARPi-FL for confocal microscopy. **B** Representative PARP1 IHC of human BE organoids. Consecutive z-stack images (step size 0.6 µm) of a representative human (**C**) or murine (**D**) organoid incubated with 0.5 µM PARPi-FL for 10 min. Green fluorescence = PARPi-FL

We used organoids derived from humans and from our L2-*IL1B* mice to determine if their PARP1 expression leads to nuclear uptake of the fluorescent imaging agent PARPi-FL. The organoid cultivation workflow, PARPi-FL staining and IHC are depicted in Fig. 2A. IHC data showed a strong PARP1 expression in the nuclei of the dysplastic epithelial cells within the patient-derived organoids (arrows; Fig. 2B). The acquired z-stacks (Fig. 2C) after incubation with PARPi-FL showed a distinct nuclear PARPi-FL accumulation in dysplastic epithelial cells as well, matching the PARP1 expression pattern.

To determine if the L2-*IL1B* mouse model exhibited similar PARP1 expression and PARPi-FL uptake as observed in human organoids, we cultured BE organoids from L2-*IL1B* mice with different age and stained them with PARPi-FL. Here, we also found nuclear PARPi-FL uptake in epithelial cells (Fig. 2D), resembling the results obtained in human organoids. Hence, we found rapid, nuclear localization of PARPi-FL in both patient-derived and murine BE organoids, indicating feasibility of PARP1 imaging for detection of the malignant transformation from BE to EAC.

### PARPi-FL localized into dysplastic lesions at the squamocolumnar junction after systemic injection in L2-*IL1B* mice

**Fig. 3:**
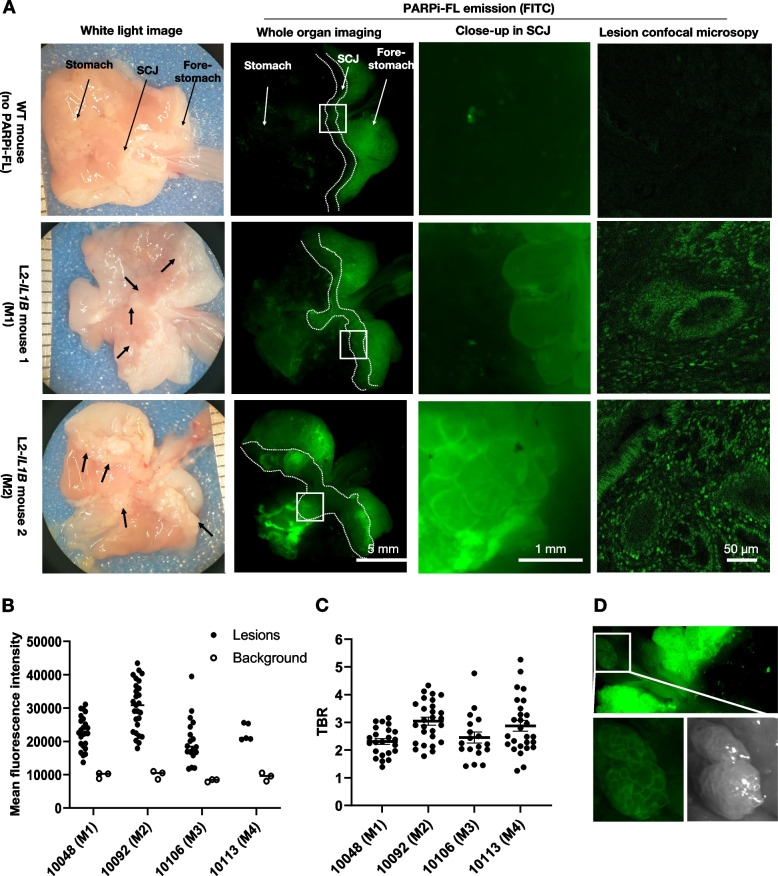
Ex vivo wide-field imaging showed PARPi-FL accumulation in dysplastic lesions at the squamocolumnar junction (SCJ) of L2-*IL1B* mice. **A** Representative white light and fluorescence ex vivo images of the whole excised stomach of a PBS-injected control (WT mouse) and two PARPi-FL injected L2-*IL1B* mice and confocal microscopy of dysplastic lesions at the SCJ. Black arrows point to macroscopically visible lesions in the L2-*IL1B *mice. White dotted lines outline the SCJ and white squares are enlarged in the close-up image. **B** Quantification of PARPi-FL-related fluorescence of all lesions at the SCJ and background fluorescence intensity of each PARPi-FL-injected L2-*IL1B* mouse (single lesions and mean plotted). **C** Tumor-to-background ratio (TBR) of individual lesions in PARPi-FL-injected animals. Data are represented as single plotted values of each lesion ROI and mean ± SEM per mouse. **D** An incidental lesion at the esophagus showed similar PARPi-FL uptake (MFI ~ 15,810) to the SCJ

After confirming high PARP1 expression in the diseased SCJ of L2-*IL1B* mice and successful uptake of PARPi-FL by human and murine organoids, a proof-of-concept in vivo experiment was performed. Four L2-*IL1B* mice, 15 to 18 months old, with large and visible dysplastic lesions, as previously reported in mice aged over 12 months [45], were i.v. injected with 75 nmol PARPi-FL and sacrificed 1 h p.i. for imaging of the whole excised stomach. Two WT mice injected with PBS instead of PARPi-FL were used as negative controls. Ex vivo imaging of the stomach showed that PARPi-FL (detected by FITC channel) accumulated in lesions at the SCJ of L2-*IL1B* mice, creating clear contrasts from surrounding tissue (Fig. 3A). Using stereomicroscopy, pronounced tissue autofluorescence was observed in the forestomach area above the SCJ. Its autofluorescent nature was apparent because it occurred not only in PARPi-FL-injected mice but also in PBS-injected WT mice, which did not have any lesions. Furthermore, the forestomach autofluorescence was not observed with other imaging modalities, i.e. IVIS imaging **(**Fig. S2) and confocal microscopy (Fig. S3). Importantly, only lesions in the SCJ were used for analysis and the forestomach area was excluded from quantification. Confocal microscopy images confirmed the PARPi-FL accumulation in cell nuclei of the dysplastic epithelium at the SCJ and the basal stratum of squamous epithelium (Fig. 3A, Fig. S3A). Quantification of the PARPi-FL signal in all macroscopically visible lesions in the SCJ showed consistently higher PARPi-FL signals in lesions compared to background, supporting feasibility of PARPi-FL-based lesion identification (Fig. 3B). This was further confirmed by the high average TBR of 2.67 ± 0.17 (Fig. 3C).

Although most lesions emerge at the SCJ, they can also appear along the distal esophagus in the L2-*IL1B* mouse model [46]. In such incidental cases, a strong PARPi-FL signal was observed in those lesions, but not in the adjacent esophagus (Fig. 3D). Indeed, confocal microscopy showed only weak accumulation of PARPi-FL in basal stratum of normal squamous epithelium (Fig. S3A-B). Via IHC, we further confirmed a high expression of PARP1 in the dysplastic SCJ areas of the PARPi-FL injected L2-*IL1B* mice and the lower PARP1 expression at the SCJ of WT (Fig. S3B) observed in our previous evaluation (Fig. 1D). Hence, systemically injected PARPi-FL localized in the late-stage lesions of L2-*IL1B* mice, allowing a clear identification of dysplastic lesions by ex vivo wide-field imaging.

### Systemic administration of PARPi-FL allowed imaging of dysplastic lesions by FME in L2-*IL1B*/*IL8*Tg mice

Following the successful ex vivo wide-field imaging of dysplastic lesions at the SCJ, we aimed to determine if PARPi-FL can also improve delineation of lesions with FME in situ. Therefore, we i.v. injected PARPi-FL in L2-*IL1B*/*IL8*Tg mice of different age groups (from 6 to 15 months old), which were fed chow (*n*= 9) or HFD (*n* = 3). The mice were sacrificed one hour p.i. and FME was performed, followed by ex vivo wide-field imaging, confocal microscopy, histopathology, and IHC. We determined the disease stage of all mice by calculating the combined score (score_comb_). We found that four out of five mice with dysplasia grade 3 also had the highest score_comb_ (score 3), while mice with dysplasia grade 2 were equally distributed among score_comb_ 1 (*n* = 3) and 2 (*n* = 3). The lowest dysplasia grade (0–1) was only found in one mouse, which also had the lowest score_comb_ (score 1). The PBS-injected L2-*IL1B*/*IL8Tg* mice (*n* = 3; 15 months old) had dysplasia grade 2 and a score_comb_ of 2. Interestingly, lesions were similarly distributed in L2-*IL1B*/*IL8Tg* mice with dysplasia grade 2 and 3, while the size of lesions was slightly different (Supplementary Tables 1–2).

**Fig. 4:**
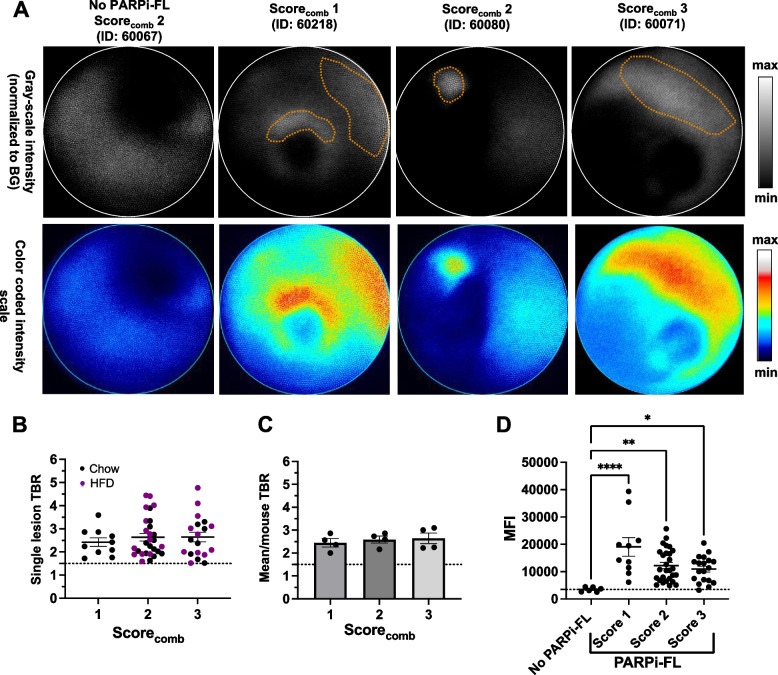
PARPi-FL enabled lesion detection in L2-*IL1B*/*IL8*Tg mice with dysplastic lesions by fluorescence molecular endoscopy (FME). **A** Representative endoscopy frames of PARPi-FL injected and non-injected mice of different scores_comb_. Gray-scale and intensity-coded false color images of selected frames are displayed. Displayed gray-scale images were normalized to the background (BG) by subtracting the average BG value from each whole image. Individual lesions are highlighted (orange dotted line). **B** Quantification of TBRs of all individually located lesions in PARPi-FL-injected mice by FME with mean ± SEM. **C** Quantification of the mean TBR of all lesions per mouse in PARPi-FL-injected mice with mean ± SEM. **D** Comparison of mean fluorescence intensity (MFI) measured in non-injected mice (*n* = 2) and the detected lesions in PARPi-FL-injected mice (*n* = 12). One non-injected mouse was excluded from the endoscopy quantification due to errors in setting acquisition parameters. In non-injected mice, the regions with the highest fluorescence detected by the endoscopy system were considered as a comparison. The quantification of the fluorescence was performed blindly. Significance levels were determined by one-way ANOVA followed by Dunnett’s multiple comparison test. * *p* < 0.05, ** *p* < 0.01 and **** *p* < 0.0001

During FME along the esophagus and the SCJ, we were able to observe lesions via clearly visible fluorescence signals in PARPi-FL-injected mice. A video of the FME in a PARPI-FL-injected mouse with a score_comb_ of 2 showed bright, localized fluorescent signals, which can be attributed to the accumulation of PARPi-FL in dysplastic lesions (Additional movie file 1). We could not identify any lesions by FME in the PBS-injected mice (Additional movie file 2). Since PARPi-FL fluoresces in the visible range of light, parallel imaging in a color channel for an overlay was not possible. Examples of FME frames in PARPi-FL- and PBS-injected mice are shown in Fig. 4A.

ROI-based quantification of all lesions detected in PARPi-FL-injected mice revealed similar TBRs (mean ± SEM) between all mice: 2.42 ± 0.18 (score_comb_ 1), 2.63 ± 0.16 (score_comb_ 2) and 2.65 ± 0.21 (score_comb_ 3; Fig. 4B). Very similar mean TBRs of 2.45 ± 0.18 (score_comb_ 1), 2.59 ± 0.15 (score_comb_ 2) and 2.64 ± 0.24 (score_comb_ 3) were found when using the mean TBR per mouse (Fig. 4C). Importantly, the observed lesions in PARPi-FL-injected mice had a significantly brighter MFI than regions with fluorescent signals in non-injected animals (Fig. 4D). These results indicate the capability of PARPi-FL-targeted FME to detect lesions in L2-*IL1B*/*IL8Tg* mice having different disease stages.

### Dysplastic lesions visible during FME were detected by wide-field imaging and confocal microscopy

**Fig. 5:**
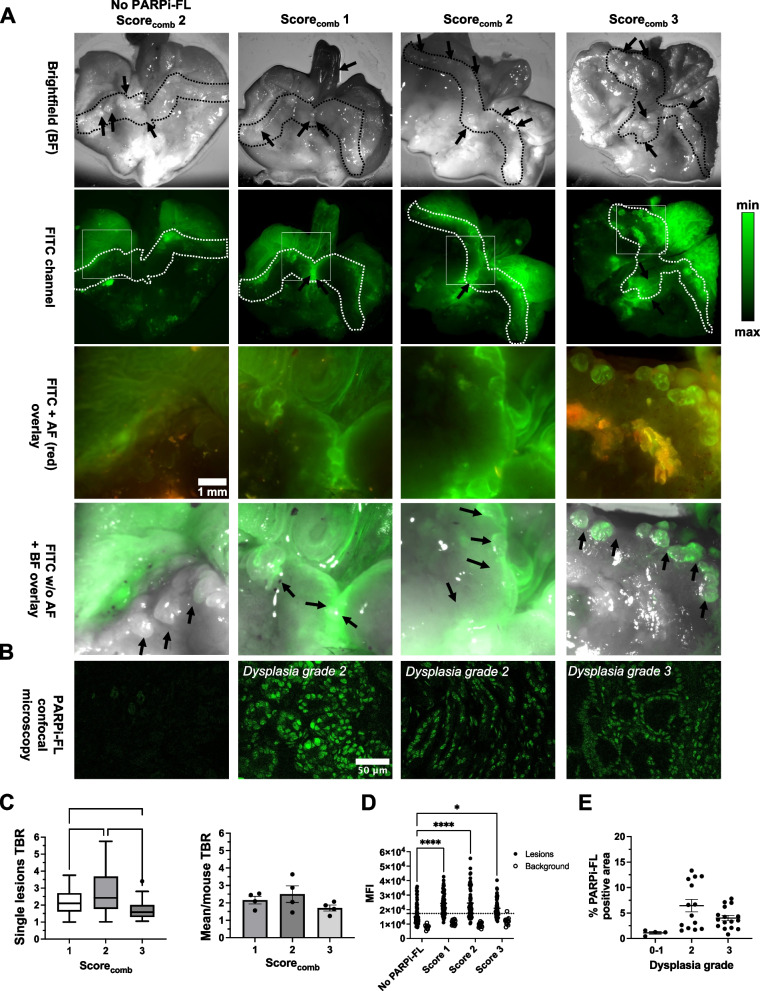
Ex vivo imaging following FME indicated that lesion detection during endoscopy in L2-*IL1B*/*IL8*Tg mice was PARPi-FL specific. **A** Ex vivo wide-field fluorescence images of excised stomachs from PARPi-FL-injected and non-injected mice that underwent FME. The SCJ is marked by dotted lines, while black arrows indicate the lesions. Only in PARPi-FL-injected mice, lesions in the brightfield (BF) were also visible in the FITC channel (arrows and close up images). **B** Confocal microscopy of the SCJ of the same mice confirmed nuclear PARPi-FL uptake. **C** Quantification of single lesions plotted per Tukey’s method with outliers and line at the median (left) and mean TBR per mouse (center) in PARPi-FL-injected mice by ex vivo wide-field imaging with mean ± SEM. ** *p* < 0.01 and **** *p* < 0.0001 by one-way ANOVA followed by Tukey’s test. **D** Mean fluorescence intensities (MFI) in macroscopically visible lesions measured in ex vivo wide-field imaging comparing non-injected with PARPi-FL injected mice. The horizontal line represented the mean MFI of PBS-injected mice. * *p* < 0.05 and **** *p* < 0.0001 by one way ANOVA followed by Tukey’s test. **E** Quantification of confocal microscopy analysis of sections at the SCJ of PARPi-FL-injected mice according to the dysplasia grade. No significant difference in PARPi-FL signal between dysplasia grade 2 and 3 was found (*p* = 0.149). Data are plotted as single values per each field and mean ± SEM (grade 0–1 = 1 mouse; grade 2 = 4 mice; grade 3 = 5 mice)

Following FME, we excised the stomachs for wide-field imaging of the SCJ, similar to the previous experiment, and calculated lesion TBRs. Indeed, macroscopically visible lesions at the SCJ, and occasionally at the esophagus, showed bright green fluorescent signals, which did not occur in the non-injected animals (Fig. 5A, arrows). Here, we also processed the images by subtracting the autofluorescence recorded in the red channel prior to creating a PARPi-FL overlay with the BF images. The overlays clearly show PARPi-FL accumulation in lesions of injected animals, but not in PBS-injected controls, despite the presence of lesions in the BF (Fig. 5A, arrows). Additionally, confocal microscopy of frozen SCJ samples showed the expected nuclear localization of PARPi-FL in all injected mice, whereas no nuclear fluorescence was observed in PBS-injected mice (Fig. 5B).

Quantification of ex vivo wide-field imaging data showed mean lesion TBRs > 2 in PARPi-FL-injected L2-*IL1B*/*IL8Tg* mice with a score_comb_ of 1 (TBR: 2.21 ± 0.08; *n* = 4) and 2 (2.71 ± 0.14; *n* = 4; Fig. 5C) and a mean lesion TBR of 1.71 ± 0.07 in mice with a score_comb_ of 3 (*n* = 4). Nevertheless, the MFI in all PARPi-FL-injected groups was significantly higher than PBS-injected mice (*p* = 0.049; Fig. 5D) and no fluorescence signal (using AF488 channel) was detected in PBS-injected mice by confocal microscopy. Finally, quantification of PARPi-FL uptake by % pta showed similar PARPi-FL signal between PARPi-FL-injected mice with dysplasia grade 2 and 3 (Fig. 5E), confirming that an increase of dysplasia grade did not result in the detection of a distinctly higher PARPi-FL signal.

PARP1 IHC of this mouse cohort also confirmed increased PARP1 expression in dysplastic epithelial cells of the SCJ (Fig. S4). Altogether, our results obtained in the L2-*IL1B* mouse model strongly suggest that the localized fluorescence signals observed by FME correspond to the PARPi-FL uptake in dysplastic cells within the SCJ lesions as further assessed by imaging after excision and confocal microscopy.

## Discussion

Novel endoscopic imaging modalities and molecularly targeted ligands for early detection of dysplasia and EAC are urgently needed to overcome the limitations of the state-of-the-art Seattle protocol, which relies on WLE and random biopsy collection. We herein report data supporting the use of PARPi-FL for FME to facilitate the identification of dysplastic and cancerous lesions that develop from non-malignant BE tissue and enable early intervention strategies based on the overexpression of PARP1.

To determine the relevance of PARP1 for the identification of dysplasia and EAC in BE patients, we demonstrate here that PARP1 expression was low in normal squamous epithelium, slightly elevated in BE without IEN but strongly increased when IEN was present. This confirms an earlier study, where increased PARP1 expression in EAC was reported in human biospecimens [33]. This indicates that PARP1 is a suitable biomarker especially for early detection of the malignant transition to EAC. The endoscopic mucosal resection samples displayed a heterogeneous mix of normal epithelium, BE without and with dysplasia and EAC lesions. PARP1 expression was significantly increased in focal areas of BE with dysplasia and EAC, indicating feasibility of localizing such pathologic areas within BE and normal esophageal tissue. This is a decisive advantage over WLE, where malignant and premalignant lesions cannot be reliably distinguished from BE areas without dysplasia based on their macroscopic visual appearance, but only based on histopathologic evaluation of a biopsy.

To date, only a few studies have explored the role of PARP1 in BE and EAC. Zhang et al. have suggested that PARP1 hyperactivation in esophageal cells caused by GERD-induced oxidative stress and PARP1-mediated activation of NF-κB pathway may contribute the pathogenesis of BE [54]. Indeed, an up-regulation of PARP1 in BE patient tissues was observed by microarray compared with normal esophageal tissue [34]. This is consistent with a higher expression of PARP1 in human BE tissues than normal esophageal epithelium in our data. The authors also demonstrated the expression of PARP1 in a surgically-induced mouse model of BE [34]. Despite the differences to our transgenic mouse model of BE with a progressive, IL-1β-driven esophagitis with dysplasia and metaplasia, in the surgically-induced model esophagitis and esophageal hyperplasia displayed a higher expression of PAR vs normal esophageal tissue, in addition to γH2AX and NF-κB [34]. We did not find any evidence indicating that PARP1 is overexpressed in other esophageal diseases in the submucosal resections examined in this study nor in the literature.

For FME and ex vivo imaging, we used the L2-*IL1B* mouse model (L2-*IL1B* and L2-*IL1B*/*IL8*Tg mice), which shows an age-dependent and IL-1β-driven progression from BE to EAC at the SCJ, recapitulating human histopathology [46, 47]. The usefulness of this model to evaluate new strategies for FME using fluorescent ligands was shown previously [43, 44]. Similar to the human biospecimens, we found increasing PARP1 expression with increasing dysplasia stage at the SCJ, with the highest expression levels associated with dysplasia grade 2 and 3. Our imaging results of pre-cancerous lesions were consistent in both the L2-*IL1B* and L2-*IL1B*/*IL8*Tg mice (with an accelerated inflammatory and dysplastic phenotype) [46] and reflect our previous data on PARPi-FL in imaging transplanted tumors derived from different human esophageal cancer lines in nude mice [33]. In addition to the animal model, the FME used in this study is a miniaturized version of an endoscope for human imaging and has been used in human studies already [55, 56]. The only differences are the diameter of the fiberscope (i.e. 0.8 mm *vs*. 2.4 mm), the detection and excitation wavelengths as the human endoscope is suited for near-infrared (NIR) imaging. Therefore, we consider our preclinical study results highly relevant and translatable to in-human imaging.

Apart from FME with biomarker-targeted probes, other novel methods for image-enhanced endoscopy, such as confocal laser endoscopy (CLE) and probe-based CLE (pCLE) have been clinically evaluated [57–59]. Generally, the success of these methods depends on highly trained users. Moreover, a recent multi-center study reporting autofluorescence imaging (AFI)-guided pCLE in 134 BE patients achieved a similar sensitivity as standard WLE for detecting dysplastic lesions, but a specificity of only 60–66.7% and false-positive rates higher than 80% [59]. Molecular markers for malignancy have the potential to increase the specificity of detection methods. For example, it has been shown that analysis of molecular markers in IHC (p53, cyclin A) can improve the diagnostic accuracy of dysplasia detection compared to the Seattle protocol [59]. Hence, in situ real-time visualization of a biomarker for dysplasia and EAC lesions achieved with a fluorescently labeled targeted probe is highly desirable to address this clinical need.

Using a miniaturized endoscope optimized for PARPi-FL detection, we were able to identify individual lesions at the SCJ during endoscopy 60 min post i.v. injection. The recorded lesions had a mean TBR of > 2.4, meaning that on average the lesion signal was 2.4-times brighter than the surrounding tissue. Interestingly, the mean TBRs per mouse were similar for all disease stages (score_comb_ 1–3). Looking at the individual lesion TBRs, we observed a slight trend towards higher TBRs in score_comb_ 2 and 3 compared to score_comb_ 1.

FME has been mostly used in combination with far-red and NIR probes [43, 44, 55, 58, 60, 61], since these wavelengths allow an overall better tissue penetration of light, display less scattering and less autofluorescence. However, PARPi-FL is constrained to the green fluorescent Bodipy-FL dye, since far red or NIR dyes significantly increase the size of the molecule to above 650 g/mol and potentially make it more hydrophilic, which will prevent efficient tissue and membrane penetration, affecting the ability of PARPi-FL to reach its nuclear target [62]. Nevertheless, the clinical utility of PARPi-FL and other green-fluorescent probes for the detection of epithelial lesions could be shown [42] and endoscopes as well as other surgical equipment for detection of PARPi-FL exist. A few other studies reported the use of green-fluorescent probes with wavelength similar to PARPi-FL. The ASY-FITC peptide, with an undisclosed target, was used for pCLE in 25 patients with a sensitivity of 75% and a specificity of 97% at a T/B ratio of 4.2 [63]. The following endoscopy study in 50 patients confirmed these values with 94% specificity and 76% sensitivity of ASY-FITC for the identification of high-grade dysplasia and EAC [64]. While promising, only histopathology is available for validation of ASY-FITC performance, but not the expression of its molecular target. Furthermore, Realdon et al. tested the feasibility of an anti-human epidermal growth factor receptor 2 (HER2) antibody labeled with AlexaFluor 488 for CLE in a surgically-induced rat model of EAC [65]. Although the fluorescence signal from EAC detected by CLE was significantly higher than BE and normal esophagus and was consistent with HER2 IHC, a high level of heterogeneity of HER2 expression within tumors was observed [65]. In contrast, PARP1 expression is usually rather homogenous in malignant lesions.

Several studies presenting phase I clinical data of FME with a targeted NIR fluorescent probes have been published in recent years, showing rapid advances in this field [55, 56, 66]. Promising data have been reported using fluorescently labeled antibodies targeting the vascular endothelial growth factor receptor A (VEGFA) and the epithelial growth factor receptor (EGFR) [55, 56]. In both studies, feasibility of detection of dysplastic lesions after topical application of Bevacizumab-800CW [55] or Cetuximab-800CW [56] in BE patients could be shown, including the detection of 25–29% of lesions that were not visible on WLE. Another study reports use of an EGFR and ErbB2 heterodimeric peptide labeled with the same dye, IR800CW [66]. In this study, high-grade dysplasia and EAC lesions showed a TBR of 1.5 in 31 patients and achieved a sensitivity and specificity of 94.1% and 92.6%, respectively [66].

In contrast to these fluorescently labeled antibodies and peptides, PARPi-FL is a lipophilic small molecule, which has the ability to penetrate tissue at a speed of 6.4 µm/s [62]. We have previously shown that both i.v. injection and local topical application of PARPi-FL are feasible application routes for epithelial cancers [33, 41, 42, 50]. While the data in the presented manuscript show tissue penetration and nuclear accumulation of PARPi-FL following i.v. injection, we have previously shown that topical application also enables rapid tissue penetration and nuclear accumulation in PARP1 expressing cells below the tissue surface, e.g. after topical application to the esophagus of a pig and after topical application to the tongue in the phase I study [33, 42]. Although we envision clinical translation of this technology with a topical application approach, we chose i.v. injection in this study since it was the first experimental approach to investigate if PARPi-FL accumulated in dysplastic lesions and enabled lesion detection in the L2-*IL1B* mouse model. I.v. application was the only available method to apply PARPi-FL in a controlled manner and in accordance with our previous experience with other optical imaging agents in this mouse model [43, 44].

Topical application in vivo in the mouse model was not feasible since there is no device to topically apply PARPi-FL in a controlled amount to the SCJ. Although oral gavage could be a potential alternative, this would result not only in local absorption in the mouse stomach but also lead to intestinal absorption and consequent probe re-distribution to target cells with a high variability between mice [67]. These technical challenges do not exist in human, where topical application in the distal esophagus is feasible through endoscopic tools and procedures.

Our approach can be further improved by addressing several study limitations. A challenge of using a green-fluorescent dye is that implementation of a simultaneous white light channel is difficult and was not available in this case. Therefore, we cannot exclude that, during endoscopy, we missed lesions that did not accumulate PARPi-FL. However, we also imaged the excised stomach of all mice and did not observe dysplastic lesions without accumulation of PARPi-FL during the evaluation of dysplastic areas at the SCJ by confocal microscopy. Another constraint of our analysis is the correlation of TBRs with PARP1 expression. The PARP1 expression was quantified in histopathology using the dysplasia grade only, while for TBR correlations we used the score_comb_ assigned to the whole excised specimen and not individual fields of view as in our previous study [44]. Additionally, we considered all the lesions developed along the SCJ for the quantification of PARPi-FL signal in the excised stomachs in contrast with the lesions only present in the cardia region and distal esophagus detected by FME. However, across all dysplasia grades, lesions could be clearly identified with the majority of individual TBRs above 2, which allows for clear lesion identification and is consistent with the expression of PARP1 at the SCJ detected by IHC of fixed stomachs. Finally, we did not perform toxicity studies after i.v. injection of 75 nmol of PARPi-FL in mice. However, our dose of PARPi-FL used in mice (1.9 mg/kg per mouse) corresponds to a human equivalent dose (HED) of 0.15 mg/kg [68]. This HED is 60-times lower than the daily clinical dose of olaparib (600 mg/day) and very unlikely to cause toxicity effects in patients.

Based on our preclinical results, we believe that clinical evaluation of PARPi-FL for early detection of both dysplasia and EAC in BE patients in a phase 1 dose escalation study is warranted. Of note, the topical application of PARPi-FL in 12 patients with oral cancer was well-tolerated with no adverse events in a phase 1 clinical trial (NCT03085147) with a tumor-to-margin ratio of > 3 at 1 µM [42], rendering PARPi-FL a very good candidate to implement FME in the surveillance of BE patients.

## Conclusion

In this study we have provided preclinical data which show the potential of the PARP1-targeted imaging agent PARPi-FL for early detection of esophageal dysplasia and cancer lesions. Using a mouse model that closely recapitulates disease progression from BE over dysplasia to EAC, we found that PARPi-FL accumulated in dysplastic lesions to a higher degree than in normal squamous and gastric epithelium, generating a clearly detectable lesion to background contrast in FME. Lesion and target specificity of this uptake was further confirmed by ex vivo wide-field imaging and confocal microscopy. Furthermore, we could show PARP1 expression in dysplastic and cancerous lesions in a small set of human BE/EAC tissue samples. Since PARPi-FL is currently evaluated in a phase 2 clinical trial for oral cancer detection after topical application, its clinical translation for early detection of dysplasia and EAC in BE patients by allowing a red-flag biopsy protocol via FME could be accelerated.

### Supplementary Information

**Additional file 1: Figure S1. **PARP1 expression in human biospecimens. Shown are representative images from patient samples illustrating the PARP1 expression in different disease stages from BE to EAC, which are often present in the same submucosal specimen. Legend: BE = Barrett’s Esophagus; EAC = esophageal adenocarcinoma; IEN = intraepithelial neoplasia.

**Additional file 2: Figure S2. **Epifluorescence imaging of whole excised stomachs (IVIS Lumina Series III). Ex vivo fluorescence imaging of a PBS-injected L2-IL1B/IL8Tg mouse, 9 months old and a PARPi-FL-injected L2-IL1B/IL8Tg mouse, 9 months old. Mice were injected with 75 nmol PARPi-FL and images were acquired 1 h post-injection by IVIS Lumina Series III (PerkinElmer, US) using the GFP filter (Ex. 500/Em. 570). Compared with the Leica M205 FCA stereomicroscope, the autofluorescence signal in the forestomach was less prominent and was not detected in the PBS-injected control. Lesions in the SCJ showed intense PARPi-FL accumulation. 

**Additional file 3: Figure S3. **PARPi-FL accumulates in the basal squamous epithelium and PARPi-FL-injected L2-*IL1B* mice express PARP1 by IHC. A) Representative confocal images of squamous epithelium and stomach from PARPi-FL-injected mice and a PBS-injected WT mouse. PARPi-FL was detected using AF488, while no AF488 signal was detected in WT mice. In PARPi-FL-injected mice, PARPi-FL uptake in normal squamous epithelium and normal gastric crypts was observed. Scale bars represent 50 μm. B) Representative IHC PARP1 images of the SCJ of the WT mouse (no dysplasia) and the two L2-*IL1B* mice (left and right, up) shown in A: PARP1 expression in dysplastic glandular epithelium of PARPi-FL-injected L2-*IL1B* mice (black arrows) was confirmed. Blue arrows indicate PARP1 + inflammatory lymphocytes. Right, down: quantification of PARP1 IHC in both WT mice and L2-*IL1B* mice (PARPi-FL-injected) confirmed the higher PARP1 expression in L2-IL1B than WT mice shown in Figure 1. PARP1 was quantified as percentage of positive area. Data are represented as single plotted values per each field (2 WT mice and 4 L2-*IL1B* mice) and mean± SEM.

**Additional file 4: Figure S4. **PARP1 expression by IHC was consistent with PARPi-FL accumulation by confocal microscopy. DAPI/PARPi-FL overlays showing PARPi-FL accumulation in nuclei of dysplastic epithelial cells at the SCJ of the PARPi-FL-injected L2-*IL1B*/*IL8*Tg mice (shown in Figure 4 and 5) by confocal microscopy. On FFPE sections of SCJ from the same mice, IHC confirmed PARP1 expression in the dysplastic lesions (blue arrows) consistent with the PARPi-FL accumulation observed by confocal microscopy. Scattered PARPi-FL-stained inflammatory lymphocytes are also present (yellow arrows).

**Additional file 5: Supplementary Table 1. **Macroscopic and microscopic scores of PARPi-FL-injected L2-*IL1B*/*IL8*Tg mice. **Supplementary Table 2. **Macroscopic and microscopic scores of non-injected L2-*IL1B*/*IL8*Tg mice.

**Additional file 6: Additional movie file 1. **Endoscopy video of a PARPi-FL-injected L2-*IL1B*/*IL8*Tg mouse. Representative endoscopy video of a L2-*IL1B*/*IL8*Tg mouse 60 minutes after the administration of 75 nmol PARPi-FL: normalized video to the global maximum pullbacks (left) and corresponding frames in logarithmic scale (right) show clearly identifiable regions with high fluorescence signal. 

**Additional file 7: Additional movie file 2. **Endoscopy video of a PBS-injected L2-*IL1B*/*IL8*Tg mouse. Representative endoscopy video of a L2-*IL1B*/*IL8*Tg mouse after the administration of PBS: normalized video to the global maximum pullbacks (left) and corresponding frames in logarithmic scale (right) don’t show any clearly recognizable area with higher fluorescence signal than surrounding esophageal tissue.

## Data Availability

The data that support the finding of this study are available from the corresponding authors on reasonable request.

## Abbreviations

PARP1: Poly (ADP-ribose) Polymerase 1
EAC: Esophageal adenocarcinoma
BE: Barrett’s Esophagus
FME: Fluorescence molecular endoscopy
WLE: White light endoscopy
OI: Optical imaging
IHC: Immunohistochemistry
SCJ: Squamocolumnar junction
IEN: Intraepithelial neoplasia
RCF: Relative Centrifugal Force
i.v.: Intravenous
p.i.: Post injection
ROI: Region of interests
TBR: Tumor-to-background ratio
NSE: Normal squamous epithelium
CLE: Confocal laser endoscopy
NIR: Near-infrared
