# Supplementary figures and images for: PARP1-targeted fluorescence molecular endoscopy as novel tool for early detection of esophageal dysplasia and adenocarcinoma
Source: J Exp Clin Cancer Res. 2024 Feb 21;43:53. doi: 10.1186/s13046-024-02963-7 (PMC10880256; doi:10.1186/s13046-024-02963-7)

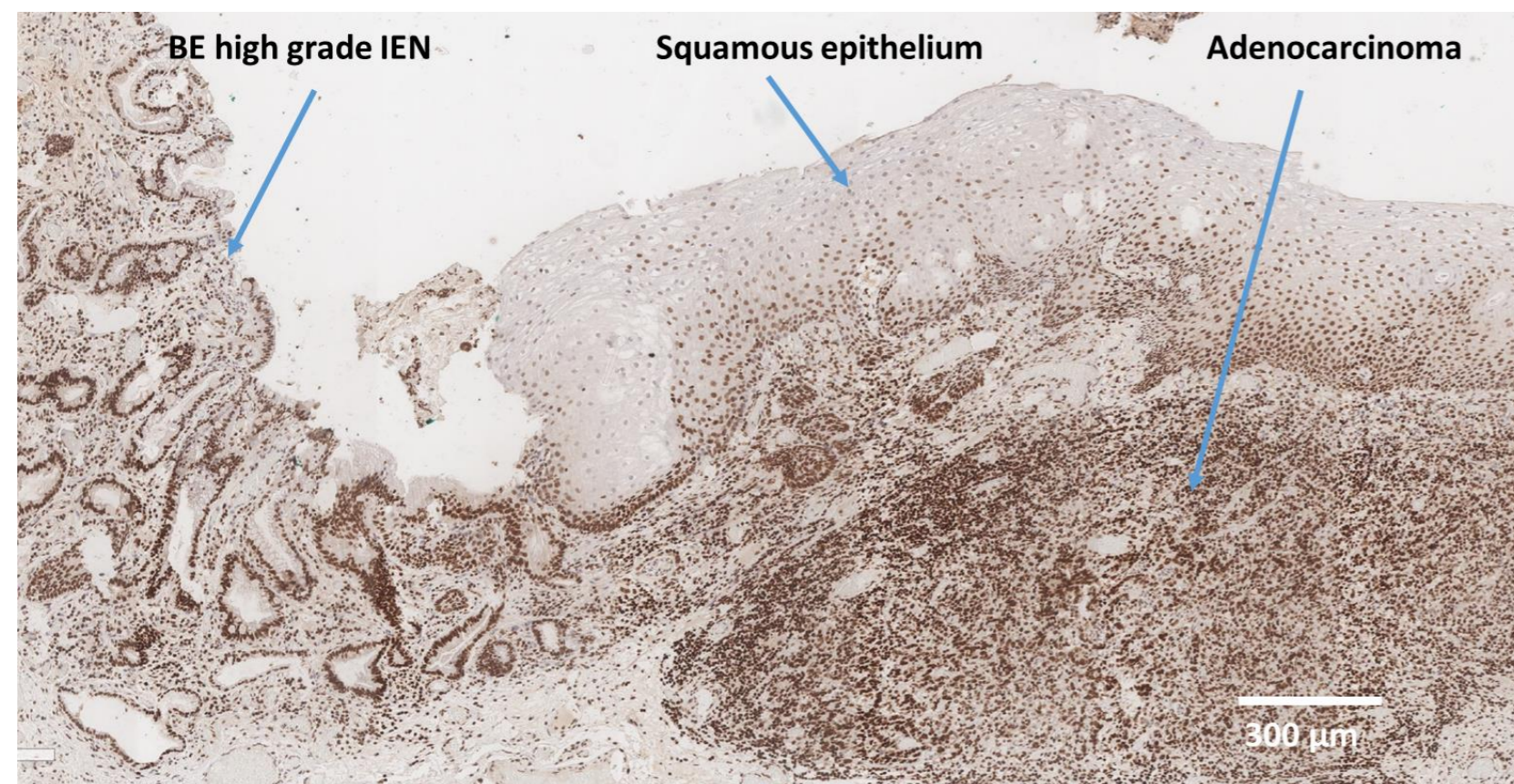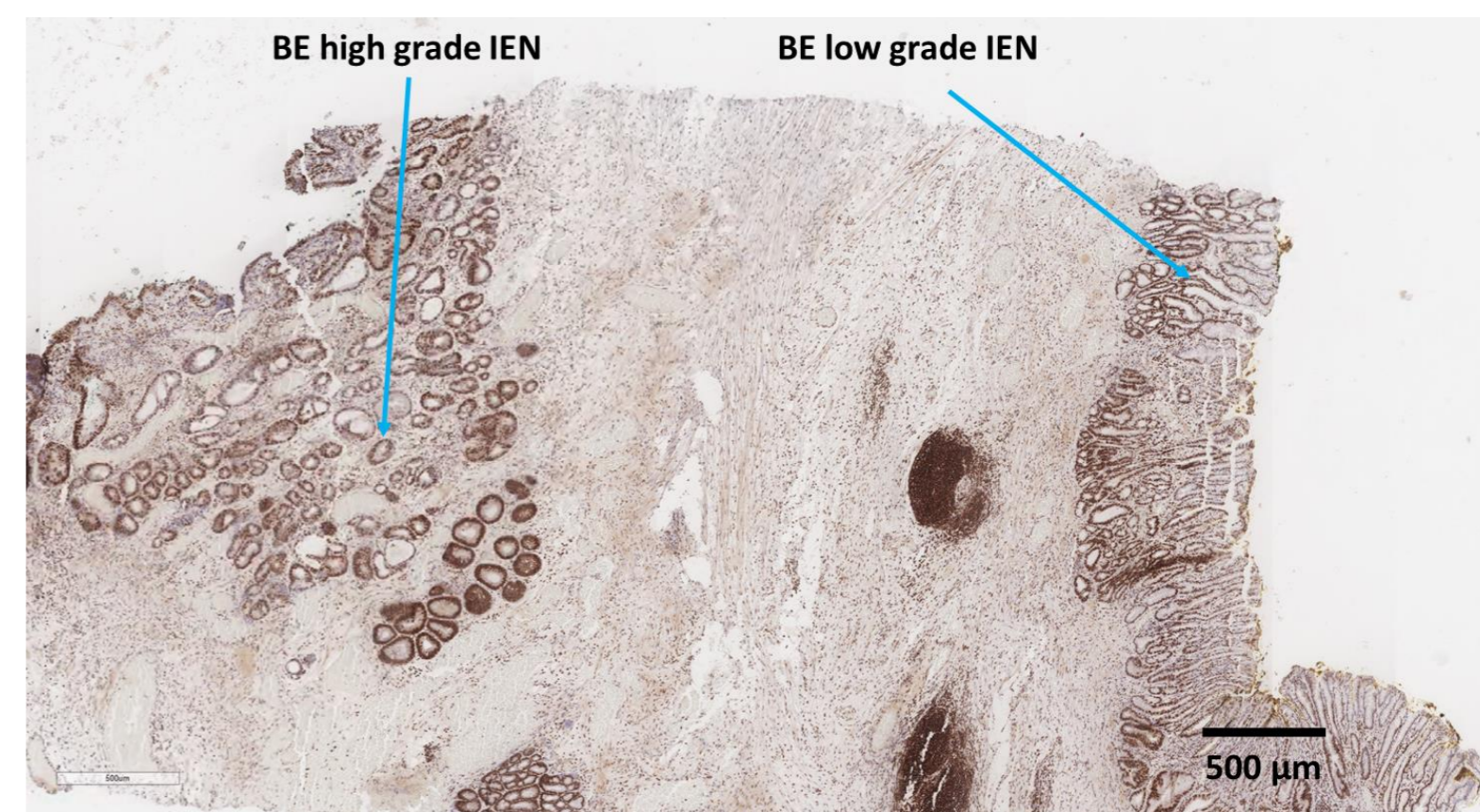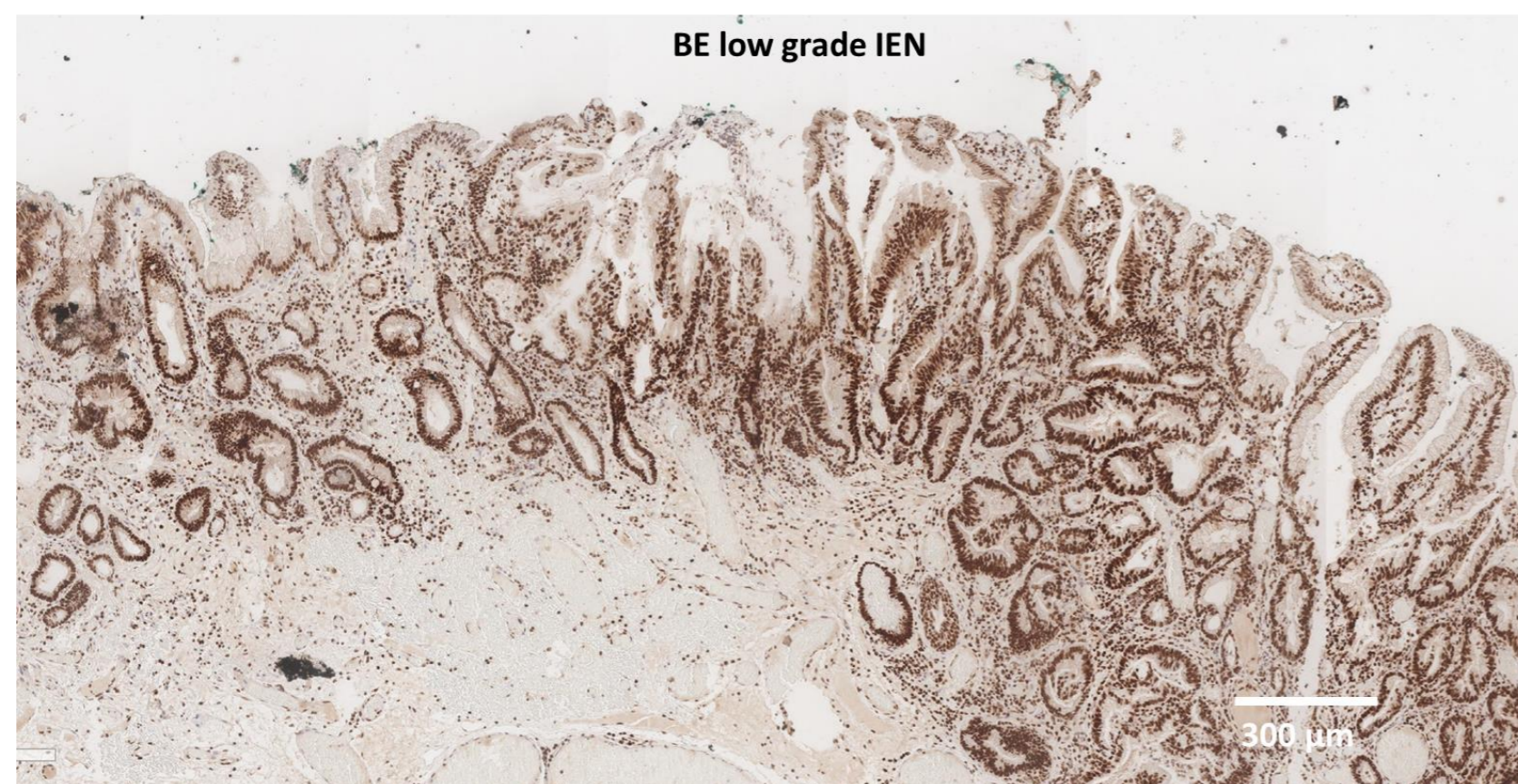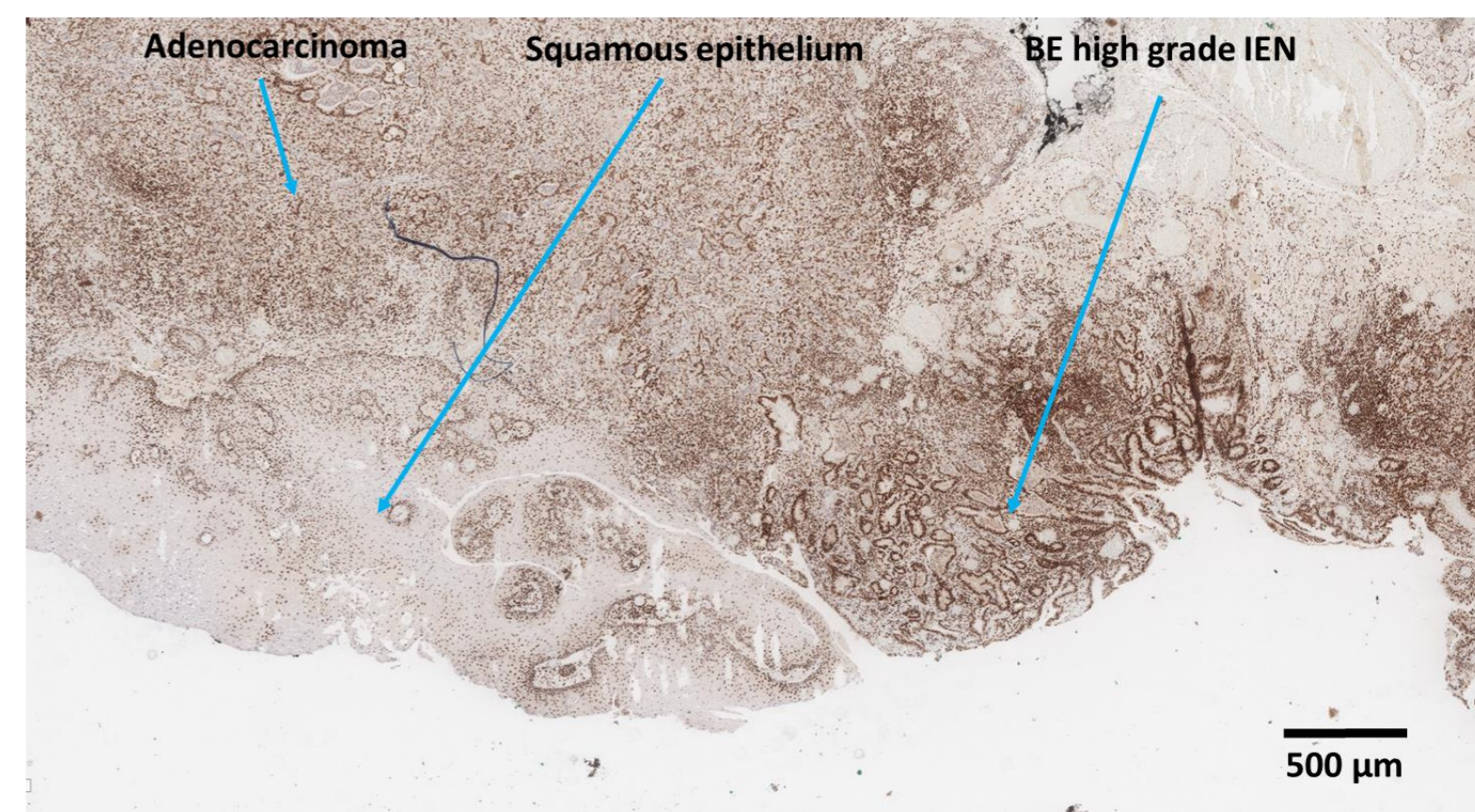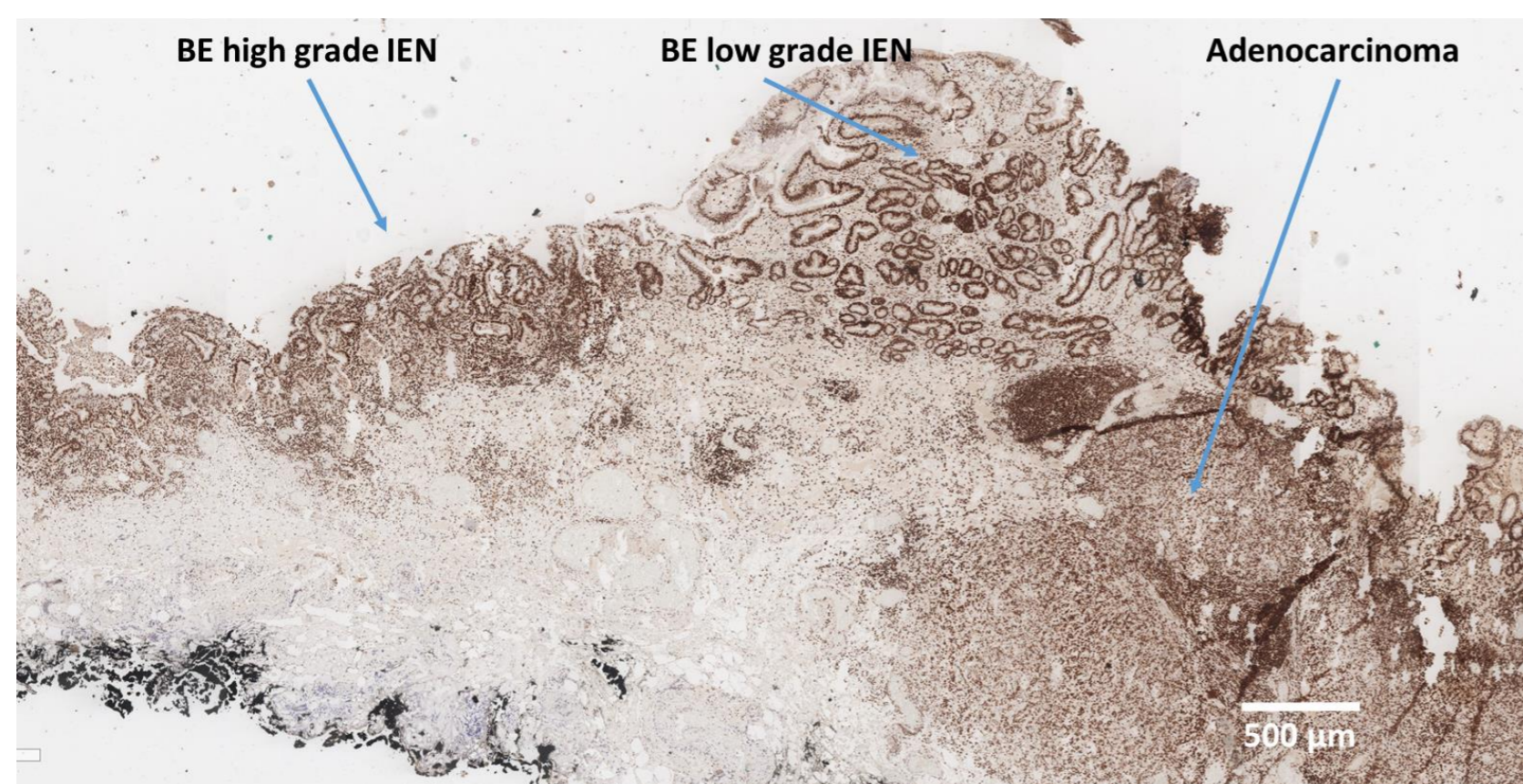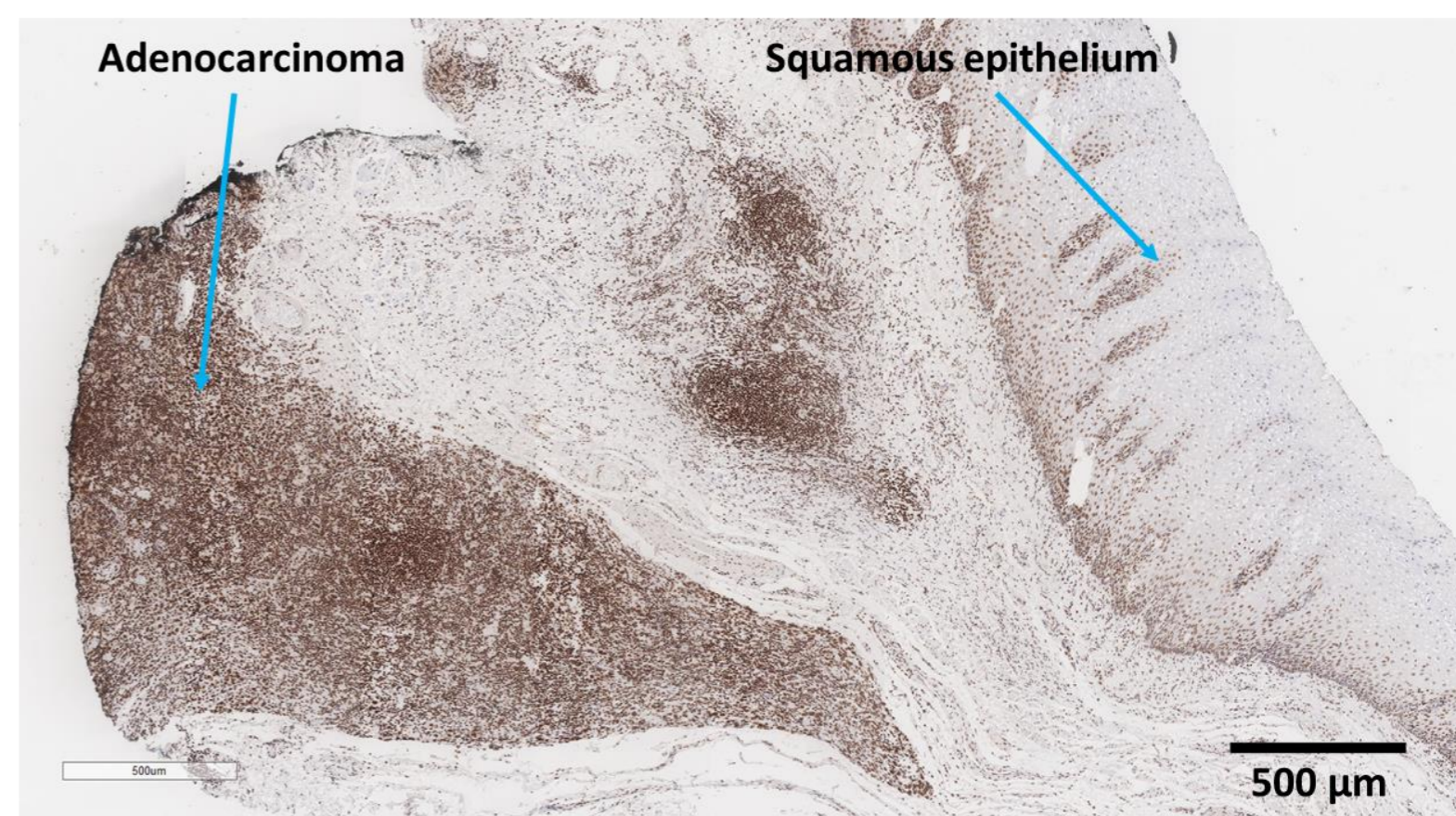

Supplement: Supplementary file 1 — Additional file 1: Figure S1. PARP1 expression in human biospecimens. Shown are representative images from patient samples illustrating the PARP1 expression in different disease stages from BE to EAC, which are often present in the same submucosal specimen. Legend: BE = Barrett’s Esophagus; EAC = esophageal adenocarcinoma; IEN = intraepithelial neoplasia. [file 13046_2024_2963_MOESM1_ESM.pdf]

**A**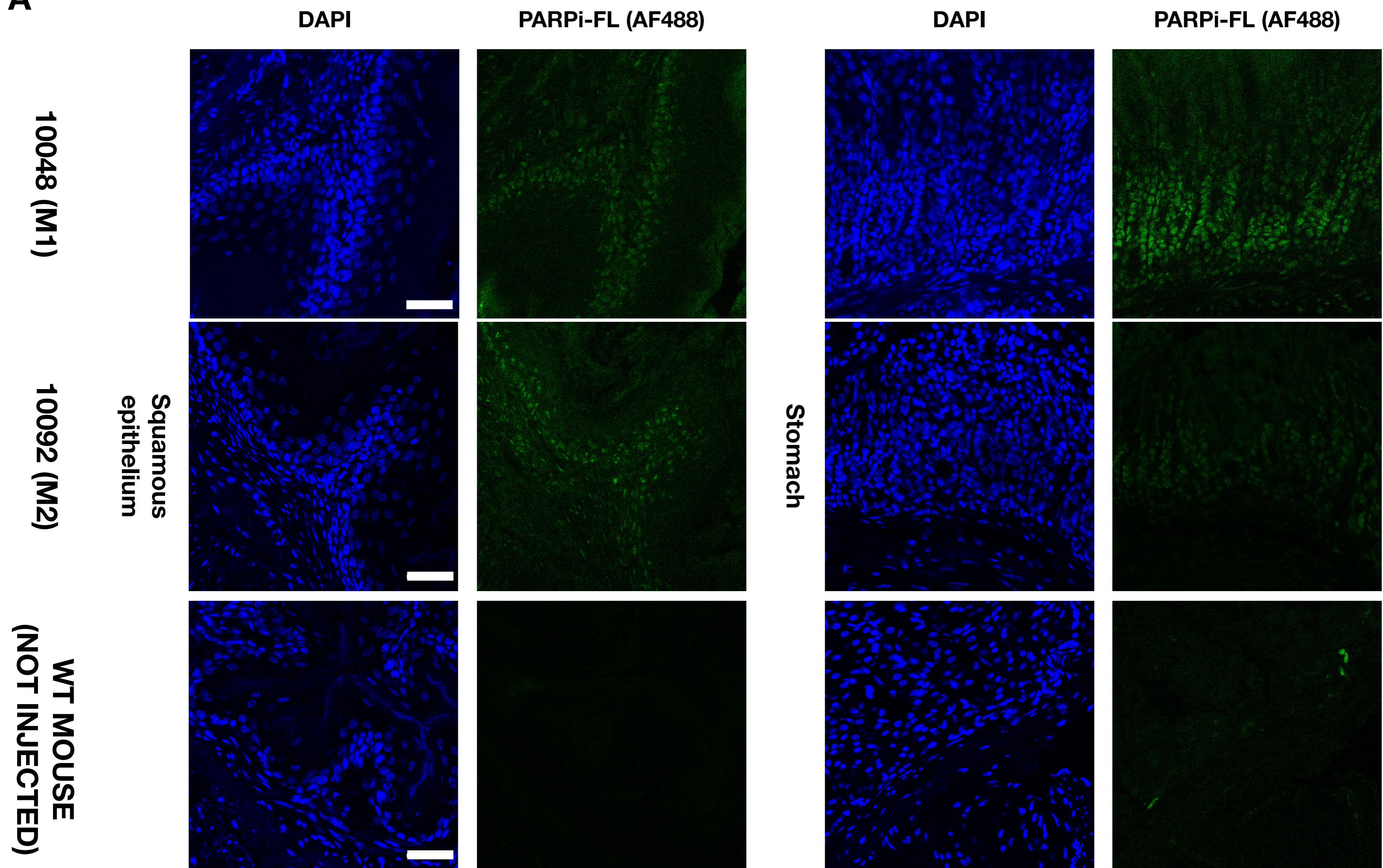**B**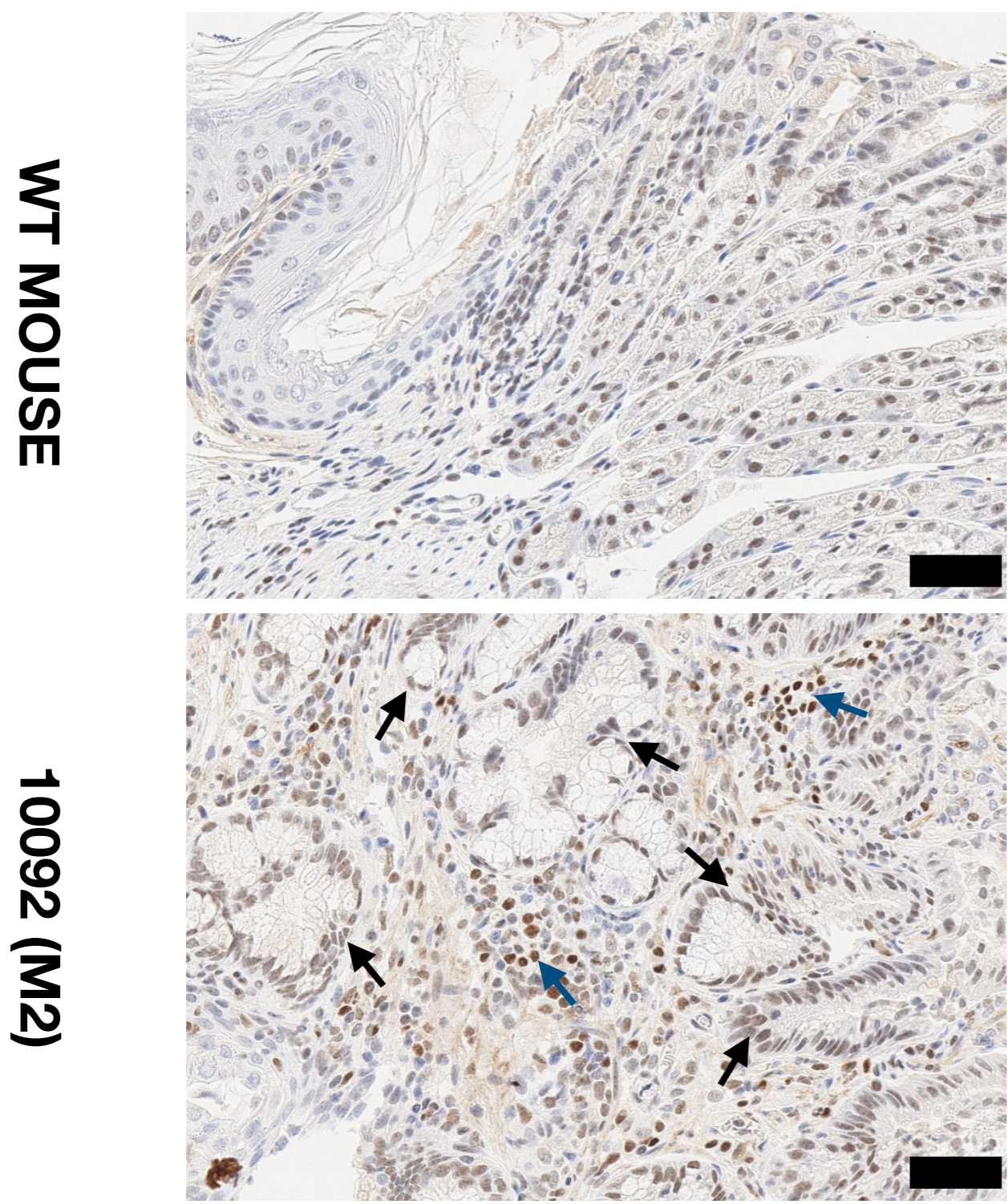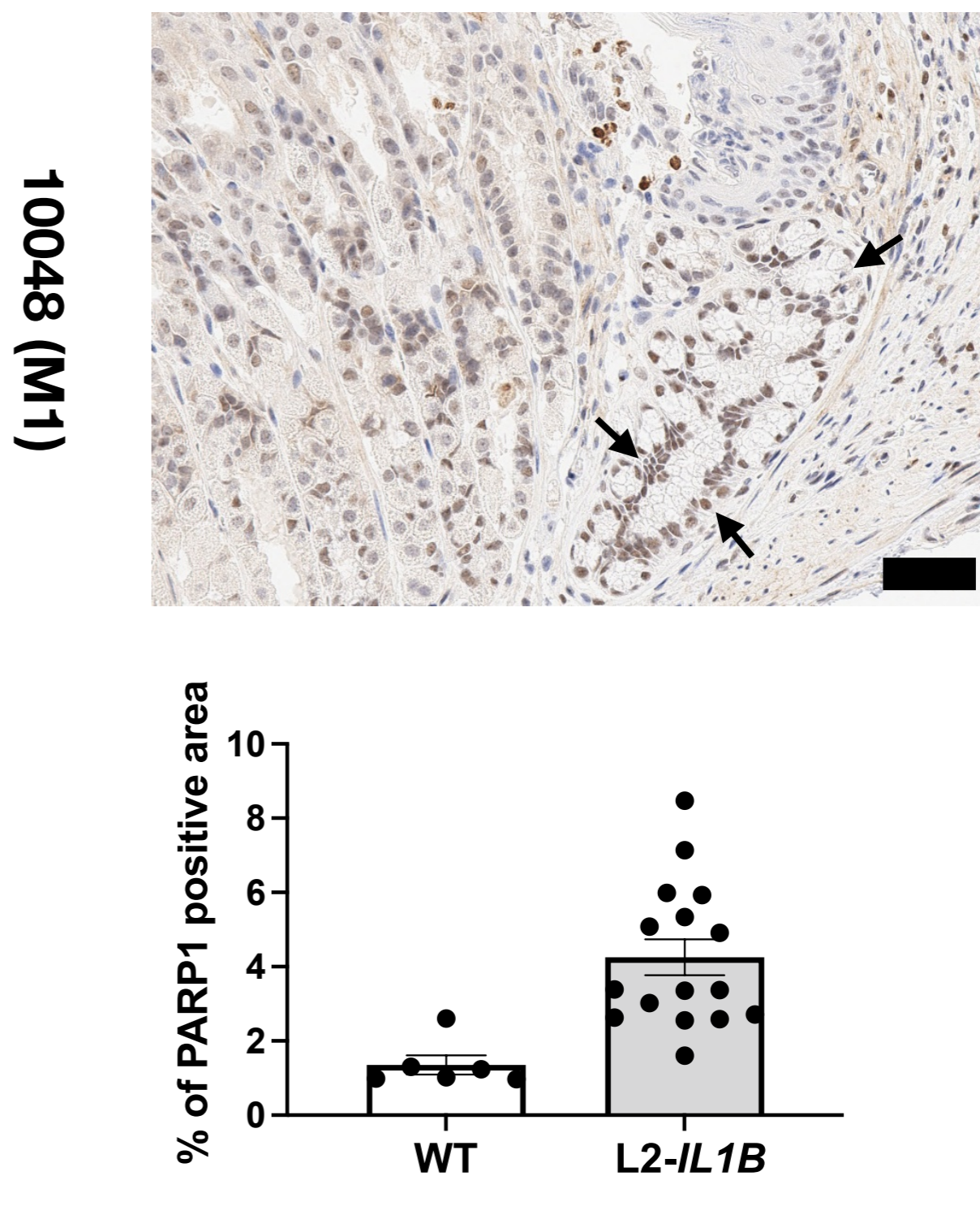

Supplement: Supplementary file 3 — Additional file 3: Figure S3. PARPi-FL accumulates in the basal squamous epithelium and PARPi-FL-injected L2-IL1B mice express PARP1 by IHC. A) Representative confocal images of squamous epithelium and stomach from PARPi-FL-injected mice and a PBS-injected WT mouse. PARPi-FL was detected using AF488, while no AF488 signal was detected in WT mice. In PARPi-FL-injected mice, PARPi-FL uptake in normal squamous epithelium and normal gastric crypts was observed. Scale bars represent 50 μm. B) Representative IHC PARP1 images of the SCJ of the WT mouse (no dysplasia) and the two L2-IL1B mice (left and right, up) shown in A: PARP1 expression in dysplastic glandular epithelium of PARPi-FL-injected L2-IL1B mice (black arrows) was confirmed. Blue arrows indicate PARP1 + inflammatory lymphocytes. Right, down: quantification of PARP1 IHC in both WT mice and L2-IL1B mice (PARPi-FL-injected) confirmed the higher PARP1 expression in L2-IL1B than WT mice shown in Figure 1. PARP1 was quantified as percentage of positive area. Data are represented as single plotted values per each field (2 WT mice and 4 L2-IL1B mice) and mean± SEM. [file 13046_2024_2963_MOESM3_ESM.pdf]
