# Supplementary material for: PARP1-targeted fluorescence molecular endoscopy as novel tool for early detection of esophageal dysplasia and adenocarcinoma
Source: J Exp Clin Cancer Res. 2024 Feb 21;43:53. doi: 10.1186/s13046-024-02963-7 (PMC10880256; doi:10.1186/s13046-024-02963-7)
Supplement: Supplementary file 2 — Additional file 2: Figure S2. Epifluorescence imaging of whole excised stomachs (IVIS Lumina Series III). Ex vivo fluorescence imaging of a PBS-injected L2-IL1B/IL8Tg mouse, 9 months old and a PARPi-FL-injected L2-IL1B/IL8Tg mouse, 9 months old. Mice were injected with 75 nmol PARPi-FL and images were acquired 1 h post-injection by IVIS Lumina Series III (PerkinElmer, US) using the GFP filter (Ex. 500/Em. 570). Compared with the Leica M205 FCA stereomicroscope, the autofluorescence signal in the forestomach was less prominent and was not detected in the PBS-injected control. Lesions in the SCJ showed intense PARPi-FL accumulation. [file 13046_2024_2963_MOESM2_ESM.pdf]

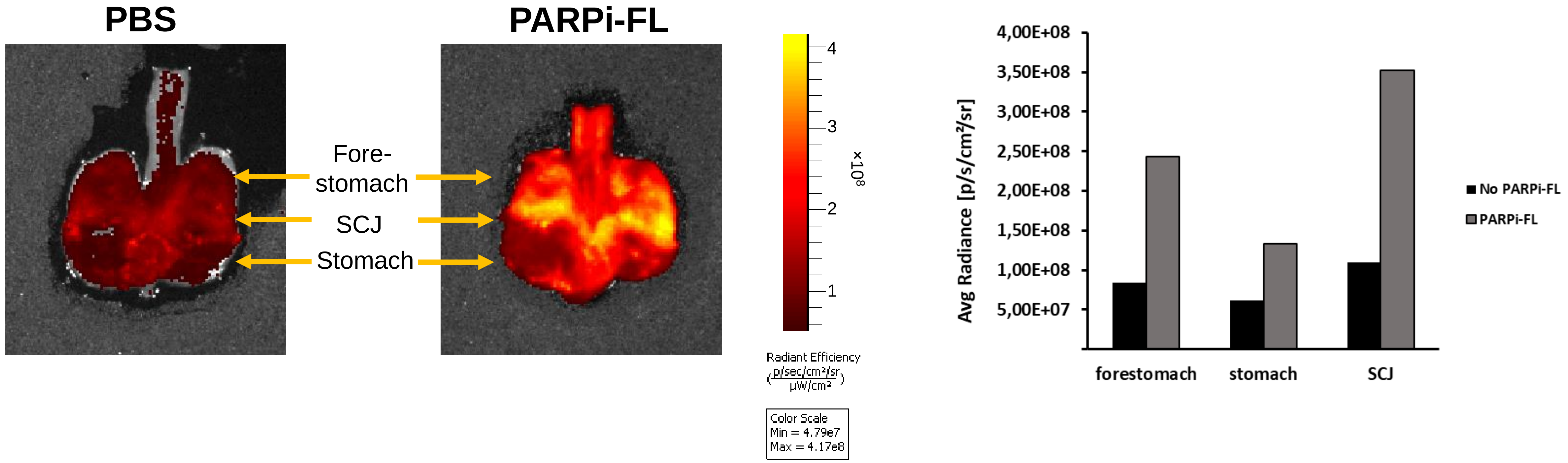

PBS
PARPi-FL
Fore-
stomach
SCJ
Stomach
4
3
2
1
×10⁸
Radiant Efficiency
(p/sec/cm²/sr / μW/cm²)
Color Scale
Min = 4.79e7
Max = 4.17e8
Avg Radiance [p/s/cm²/sr]
4,00E+08
3,50E+08
3,00E+08
2,50E+08
2,00E+08
1,50E+08
1,00E+08
5,00E+07
forestomach
stomach
SCJ
No PARPi-FL
PARPi-FL
