# Supplementary material for: PARP1-targeted fluorescence molecular endoscopy as novel tool for early detection of esophageal dysplasia and adenocarcinoma
Source: J Exp Clin Cancer Res. 2024 Feb 21;43:53. doi: 10.1186/s13046-024-02963-7 (PMC10880256; doi:10.1186/s13046-024-02963-7)
Supplement: Supplementary file 4 — Additional file 4: Figure S4. PARP1 expression by IHC was consistent with PARPi-FL accumulation by confocal microscopy. DAPI/PARPi-FL overlays showing PARPi-FL accumulation in nuclei of dysplastic epithelial cells at the SCJ of the PARPi-FL-injected L2-IL1B/IL8Tg mice (shown in Figure 4 and 5) by confocal microscopy. On FFPE sections of SCJ from the same mice, IHC confirmed PARP1 expression in the dysplastic lesions (blue arrows) consistent with the PARPi-FL accumulation observed by confocal microscopy. Scattered PARPi-FL-stained inflammatory lymphocytes are also present (yellow arrows). [file 13046_2024_2963_MOESM4_ESM.pdf]

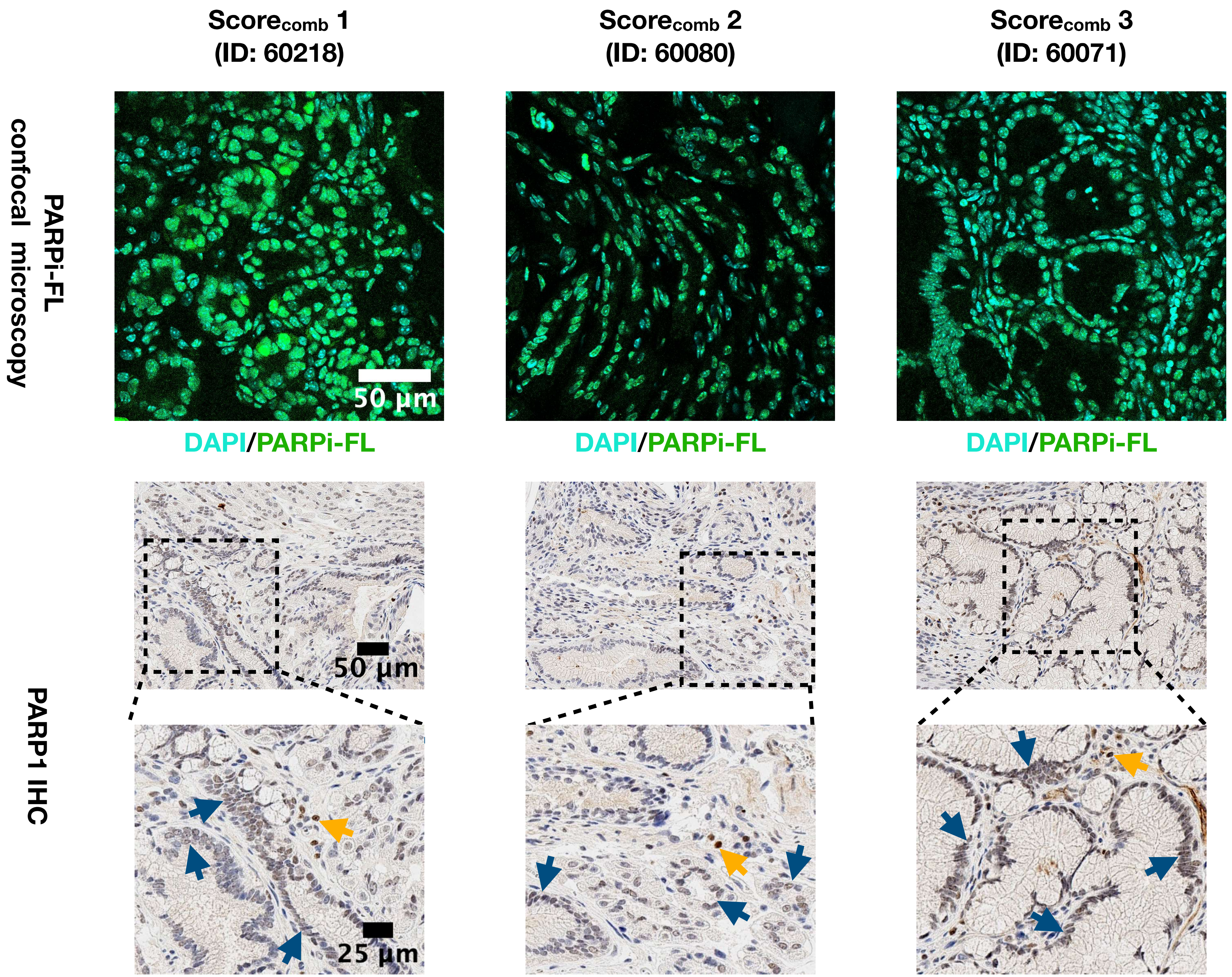
Score$_{comb}$ 1
(ID: 60218)
Score$_{comb}$ 2
(ID: 60080)
Score$_{comb}$ 3
(ID: 60071)
PARPi-FL
confocal microscopy
50 µm
DAPI/PARPi-FL
DAPI/PARPi-FL
DAPI/PARPi-FL
PARP1 IHC
50 µm
25 µm
