# Supplementary material for: PARP1-targeted fluorescence molecular endoscopy as novel tool for early detection of esophageal dysplasia and adenocarcinoma
Source: J Exp Clin Cancer Res. 2024 Feb 21;43:53. doi: 10.1186/s13046-024-02963-7 (PMC10880256; doi:10.1186/s13046-024-02963-7)
Supplement: Supplementary file 5 — Additional file 5: Supplementary Table 1. Macroscopic and microscopic scores of PARPi-FL-injected L2-IL1B/IL8Tg mice. Supplementary Table 2. Macroscopic and microscopic scores of non-injected L2-IL1B/IL8Tg mice. [file 13046_2024_2963_MOESM5_ESM.docx]

| **Mouse ID** | **Cardia coverage score (0-4)** | **Cardia tumor size (0-4)** | **Mean of macroscopic score** | **Dysplasia grade**  **(0-4)** | **Mean dysplasia grade + macroscopic score** | **Inflammation score**  **(0-3)** | **Metaplasia**  **Score**  **(0-3)** |
| --- | --- | --- | --- | --- | --- | --- | --- |
| 60071 | 2.5 | 2 | 2.25 | 3 | 2.6 | 3 | 3 |
| 60221 | 2.5 | 2 | 2.25 | 3 | 2.6 | 3 | 3 |
| 60198 | 3 | 2 | 2.5 | 3 | 2.8 | 3 | 3 |
| 60197 | 2.5 | 2 | 2.25 | 3 | 2.6 | 3 | 3 |
| 60220 | 2 | 1 | 1.5 | 3 | 2.3 | 3 | 3 |
| 60064 | 3 | 2 | 2.5 | 2 | 2.3 | 3 | 3 |
| 60080 | 3 | 1.5 | 2.25 | 2 | 2.1 | 3 | 3 |
| 60210 | 3 | 2.5 | 2.75 | 2 | 2.4 | 3 | 3 |
| 60217 | 2.5 | 1.5 | 2 | 2 | 2.0 | 3 | 3 |
| 60194 | 2 | 2 | 2 | 2 | 2.0 | 3 | 3 |
| 60218 | 2 | 2 | 2 | 2 | 2.0 | 3 | 3 |
| 60068 | 3 | 2 | 2.5 | 0-1 | 1.5 | 2 | 3 |

**Supplementary Table 1. Macroscopic and microscopic scores of PARPi-FL-injected L2-IL1b/IL8 mice**

**Supplementary Table 2. Macroscopic and microscopic scores of non-injected L2-IL1b/IL8 mice**

| **Mouse ID** | **Cardia coverage score (0-4)** | **Cardia tumor size (0-4)** | **Mean of macroscopic score** | **Dysplasia grade** | **Mean dysplasia grade + macroscopic score** | **Inflammation score** | **Metaplasia**  **Score** |
| --- | --- | --- | --- | --- | --- | --- | --- |
| 60073 | 2.5 | 2 | 2.25 | 2 | 2.1 | 3 | 3 |
| 60070 | 3 | 2 | 2.50 | 2 | 2.3 | 3 | 3 |
| 60067 | 3 | 2 | 2.50 | 2 | 2.3 | 3 | 3 |
